# Acknowledgement to Reviewers of *Sensors* in 2014

**DOI:** 10.3390/s150101106

**Published:** 2015-01-09

**Authors:** Sensors Editorial Office

**Affiliations:** MDPI AG, Klybeckstrasse 64, CH-4057 Basel, Switzerland

The editors of *Sensors* would like to express their sincere gratitude to the following reviewers for assessing manuscripts in 2014:

Abancó, Clàudia
Abbas, Ash Mohammad
Abdel-Hafez, M.F.
Abdel-Motaleb, Ibrahim M.
Abdulhalim, Ibrahim
Abich, Julian
Abollino, Ornella
Abot, Jandro
Aboutanios, Elias
Abramo, Antonio
Abramovich, Haim
Abrudan, Traian
Abuali, Najah
Abu-Mahfouz, Issam
Acosta-Jiménez, Antonio J.
Acuna, Guillermo
Adibi, Sasan
Adley, Catherine
Aeron, Shuchin
Afroozeh, Abdolkarim
Afzal, Adeel
Agafonov, Vadim
Aggelis, Dimitri
Aggelis, Dimitrios G.
Agili, Sedig S.
Agudo, J.
Aguirre-Salado, Carlos Arturo
Ahmad, Manzoor
Ahmad, Iftekhar
Ahn, JungHo
Ai, Huiwang
Ai, Shiyun
Aiello, Orazio
Aillerie, Michel
Akca, Devrim
Akhloufi, Moulay A.
Aksakal, Sultan Kocaman
Al-Anbagi, Irfan S.
Alander, Jarmo
Alanis, Arnulfo
Albiol, Alberto
Albiol, Sergio
Albrecht, Bettina
Alenyà, Guillem
Alghooneh, Mansoor
Ali, Abdelrahman
Aliakbarpour, Hadi
Ali-Löytty, Simo
Allegretti, Ana
Al-Manasir, Khalil
Almeida, Aitor
Al-Naemi, Faris
Alnusair, Awny
Aloisi, Giovanni
Alonso, Eduardo
Alonso, Luis
Alonso-Lomillo, M. Asunción
Al-Sarawi, Said F.
Al-Sousy, Fayez F. M.
Altdorff, Daniel
Altet, Josep
Altucci, Carlo
Alvarez, Jose M.
Alvarez-Lorenzo, Carmen
Ambrosini, Dario
Amditis, Angelos
Amft, Oliver
Amiruzzaman, Md
Amor, Mercedes
An, Beongku
Anderson, Alan
Anderson, Sharolyn
Andrade-Cetto, Juan
André, Nicolas
Andreev, Sergey
Andrei, Octavian
Andreoni, Giuseppe
Andújar, Dionisio
Andujar, Jose Manuel
Anfossi, Laura
Angelidis, Pantelis A.
Ania-Castañon, Juan Diego
Annamdas, Venu
Annamdas, Venu Gopal Madhav
Antfolk, Christian
Anthony, Carl J.
Antona, Margherita
Antończak, Arkadiusz
Antonopoulos, Angelos
Anvar, Amir Mohammad
Aoki, Hiroshi
Apetrei, Constantin
Apostolova, Emilia L.
Appiah, Kofi
Arana, Sergio
Araújo Godinho, Maria Isabel
Araujo, Alvaro
Arbault, Stephane
Arca, Francesco
Arduini, Fabiana
Arena, Antonella
Arfaoui, Ahlem
Arganda-Carreras, Ignacio
Argus, Donald
Argyriou, Antonios
Ariga, Katsuhiko
Ariño, Cristina
Ariyur, Kartik
Arjunan, Sridhar Poosapadi
Arlt, Volker M
Armenakis, Costas
Armingol, Jose Maria
Arnold, Mark A.
Arribas, Javier
Arriola, A
Arrue, Jon
Artese, G.
Artola, Adriana
Artz, Thomas
Arya, Sunil
Asensi, Marise D.
Ashok, Pradeepkumar
Aslan, Kadir
Asorey-Cacheda, Rafael
Assuncao, Antonio
Assunção, Pedro
Asteriadis, Stylianos
Astráin Escola, José Javier
Atalay, Ozgur
Atallah, Louis
Atchison, Jennifer
Attivissimo, Filippo
Aubert, Hervé
Auguin, Michel
Austin, Daniel
Avolio, Alberto P
Avvenuti, Marco
Aw, K.C.
Awrangjeb, Mohammad
Ay, Chyung
Ayana, Essayas K.
Aydin, Ilhan
Azenha, Miguel
Aziz, Muhammad Zaheer
Azman, Amelia Wong
Baca, Arnold
Bach, Dominik R.
Bae, Hyungdae
Baguet, Sebastien
Bahreyni, Behraad
Bai, Meng-Yi
Bai, Xiangzhi
Baig, Zubair Ahmed
Baik, Sung Wook
Bailey, Donald
Bailey, Ryan C.
Bajo, Javier
Bakr, Bilal Abu
Bakri-Kassem, Maher
Baktir, Selcuk
Balan, Mugur C.
Balandin, Alexander A.
Balas, Costas
Baldwin, R.P.
Balleri, Alessio
Banasik, Miroslaw
Bando, Takehiko
Bandugula, Vishal
Banerjee, Pratik
Bang, Ole
Banica, Florinel-Gabriel
Bank, Craig E.
Banno, Atsuhiko
Baños, Oresti
Bao, Guanqun
Baraban, Larysa
Baradarani, Aryaz
Barambones, Oscar
Baran, Derya
Barber, Ramon
Barone, Umberto
Barr, Chris
Barralon, Pierre
Barreira, Eva
Barrett, Gary
Barrientos, Antonio
Barron, Olga
Barshan, Billur
Barsocchi, Paolo
Bartlett, Rebecca
Bartsch, Heike
Barzaghi, Riccardo
Barzi, Emanuela
Bashkatov, Alexey Nikolaevich
Baskoutas, Sotirios
Bastide, Remi
Batchelor-McAuley, Christopher
Battie, Yann
Batur, C.
Bauer, Martin
Baumann, Peter
BAZAEI, ALI
Beauchamp, Jonathan
Becerril-Garcia, Hector A.
Beck, Olof
Begg, Rezaul K.
Begum, Shahina
Beheim, Glenn M.
Beheshti, Soosan
Bekiaris, Evangelos
Belabbas, Boubeker
Belahcen, Anouar
Bell, Scott
Bellavista, Xiao-Quan
Belle, Ashwin
Bellido, Francisco J.
Bender, Paul
Bennett, Bradford C.
Bereta, Michał
Bergasa, Luis M.
Berglin, Lena
Bergmann, Jeroen H. M.
Bernardos, Ana M
Bernards, Matt
Bernasconi, Andrea
Berneschi, Simone
Bernhard Budaker, Bernhard
Bernini, R.
Berthouze, Nadia
Bertrand, Pierre R.
Bertsch, Arnaud
Besada, Juan A.
Besada-Portas, Eva
Bestak, Robert
Bevacqua, Martina
Bevan, George
Bevly, David M.
Beyerer, Jürgen
Beyrau, Frank
Bhattarai, Keshav
Bhatti, Asim
Bhatti, Pamela
Bian, Kaigui
Biancalana, Claudio
Bien, Franklin
Bigler, Emmanuel
Bill, Ralf
Bilro, Lúcia
Birch, David M.
Birdsall, Nigel J. M.
Bishop, Greg
Bitelli, Gabriele
Bitzer, Phillip
Björninen, Toni
Blackburn, Steven
Blackman, Chris
Blanc, Philippe
Blanco, Carlosr Del
Blanco, Jose Luis
Bloisi, Domenico
Blondeau, Pascal
Bloom, Marc
Blümich, Bernhard
Bocca, Maurizio
Boedecker, Gerd
Boero, Cristina
Boesel, Luciano
Bogdanowicz, Robert
Boggia, Gennaro
Bohndiek, Sarah E
Boissy, Patrick
Bond, Alan M.
Bonhommeau, Sébastien
Bonin-Fon, Francisco
Bonizzoni, Edoardo
Bonkat, Gernot
Bonnet, Vincent
Boochs, Frank
Borges, Luís Miguel Moreira
Borisov, S.M.
Borrmann, Dorit
Bortnowska, Grażyna
Boschetti, Giovanni
Boss, Emmanuel
Bouchard, Louis
Bouganis, Christos-Savvas
Boukadoum, Mohammad R. K.
Bourbakis, Nikolaos G.
Bourdin, Pierre
Bourhis, Guy
Bouroche, Melanie
Boutejdar, Ahmed
Boutelle, Martyn G.
Bouvet, Marcel
Bouzouane, Abdenour
Bowen, Chris
Bowkurt, Alper
Bozoki, Zoltan
Brad, Remus
Bradu, Adrian
Braga, Guilherme S.
Brand, Jaco
Brand, Serge
Brandl, M.
Bratov, Andrey
Brauns, E.
Bravo, Jose
Breckon, Toby
Bremond, Francois
Brennan, Robert W.
Brenner, Claus
Brenner, Gunther
Broche, Lionel
Broderick, Patricia A.
Brodie, Matthew
Brolly, Matthew
Brooke, John M.
Brooker, Graham M.
Bröring, Arne
Brown III, Donald R.
Brown, Jeremy
Brownlee, Sandy
Bruder, Gerd
Bruggi, Matteo
Brujic-Okretic, Vesna
Brunelli, D.
Bruneo, Dario
Brunetti, Luciano
Brunner, Andreas J.
Brusey, James
Brutti, Alessio
Brzozka, Zbigniew
Bu, Xianhe
Buchowski, Maciej S.
Bucksch, Alexander
Buck-Sorlin, Gerhard
Budgett, David
Bui, Nicola
Buividas, R.
Buonaurio, Roberto
Burkart, Andreas
Burleigh, Scott
Burns, Brendan
Burrows, Susan
Büttgenbach, Stephanus
C Jones, Erick
C. M. Carvalho, Paulo
Cabeza, Rafael
Cablk, Mary E.
Cabri, Giacomo
Cahalane, Conor
Cahill, Brian P.
Cai, Shuowei
Cai, Yaqi
Cai, Yinghao
Cai, Yunze
Cai, Zhipeng
Cai, Zhongyu
Calafate, Carlos Miguel
Caldarelli, Stefano
Calders, Kim
Caleffi, Marcello
Caliendo, Cinzia
Calleja, M.
Caltenco, Héctor A.
CALVO, Angela
Calzada, M. Lourdes
Cammarano, Andrea
Campbell, Carlene
Camplani, Massimo
Campo, L Torralbo
Canning, John
Cano-Barrita, Prisciliano F. de J.
Canuto, Enrico
Cao, Hung
Cao, Kai
Cao, Xia
Cao, Zhuo
Cao, Zongjie
Capaldo, Paola
Capella Hernández, Juan Vicente
Capi, Genci
Cappiello, Cinzia
Carlen, Edwin T.
Carlone, Pierpaolo
Carlos, Juan Martína
Carmo, João Paulo Pereira Do
Carotenuto, Riccardo
Carpino, G.
Carrascosa, Laura Garcia
Carrera, Erasmo
Cartwright, Michael S.
Carvalho, Paolo
Casado, Rafael
Casale, Pierluigi
Casalicchio, Emiliano
Casbeer, David W.
Cash, Kevin
Cassidy, John
Casson, Alexander J.
Castrillón-Santana, Modesto
Catana, Ciprian
Catarino, Ana
Cavallini, Andrea
CAVALLO, Filippo
Cavicchioli, Andrea
Cayuela, J.A.
Cazorla, Miguel
Ceamanos, Xavier
Cecotti, Hubert
Cennamo, Nunzio
Cerbo, Rosa Maria
Cerri, Pietro
Cetin, Arif Engin
Chaaraoui, Alexandros André
Chai, (Raullen) Qi
Chalhoub, Gerard
Chamam, Ali
Chan, Adrian D. C.
Chan, Chia-Tai
Chan, James W.
Chan, Kit Yan
Chan, Thomas
Chan, Yang-Hsiang
Chan, Yung-Kuan
Chandler, Josephine R.
Chandler, Josie
Chang, Cheng-Chun
Chang, Chih-Wei
Chang, Chih-yuan
Chang, Fuh-Yu
Chang, Honglong
Chang, Hwan-You
Chang, I-Cheng
Chang, Jiyul
Chang, Pei-Zen
Chang, Ray-I
Chang, Rwei-Ching
Chang, Shoou-Jinn
Chang, Yao-Jen
Chang, Yia-Chung
Chang, Yuan-Jen
Chantem, Tam
Chao, Daihong
Chapeleau, X.
Chapman, Eli
Charfuelán, Marcela
Charles, Toth
Charlot, Benoît
Chartrand, Rick
Chau, Duc Phu
Chau, Lai-Kwan
Chau, Yuen
Chaudhary, Ujwal
Chegel, Vladimir
Chellappa, Rama
Chen, Bor-Sen
Chen, Chang-Shi
Chen, Chao
Chen, Chen
Chen, Chen-Yuan
Chen, Chuin-Shan
Chen, Chun-Hua
Chen, Deji
Chen, Der-Chin
Chen, Fangjiong
Chen, Fu-Kun
Chen, Gang
Chen, Haifeng
Chen, Hao
Chen, Hong
Chen, Hongyun
Chen, Hsin
Chen, Hung-Cheng
Chen, I-Ming
Chen, Jiajun
Chen, Jianhua
Chen, Junping
Chen, Kai-ying
Chen, Kun-Neng
Chen, Lieu-Hen
Chen, Lung-Chien
Chen, Mingli
Chen, Nengcheng
Chen, P.H.
Chen, Peng
Chen, Qiang
Chen, Rong-Sheng
Chen, Shih-Lun
Chen, Wei
Chen, Weimin
Chen, Xianfeng
Chen, Xiaofei
Chen, Xiaojian
Chen, Xiyuan
Chen, Xuedong
Chen, Xuemin
Chen, Xun
Chen, Yen-Da
Chen, Yenlin
Chen, Yong-Sheng
Chen, Yuanzhu
Chen, Yue
Chen, Yuwei
Chen, Zhencheng
Chen, Zhihao
Chenele, David
Cheng, Chi-Tsun
Cheng, Dengfa
Cheng, Hong
Cheng, Ming-Cheng Mark
Cheng, Ming-Yuan
Cheng, Shunfeng
Cheng, Tao
Cheng, Teng
Cheng, Wei Chen
Cheng, Weiying
Cheng, Wood-Hi
Cheng, Xiuzhen
Cheng, Xuanhong
Cheong, Kai Loon
Chi, Kuang-Hui
Chiabrando, F.
Chiang, Kai-Wei
Chiang, Yi-Ting
Chiappini, Ciro
Chiesa, Maria
Chih, Mingchang
Chin, Craig A.
Chiolerio, Alessandro
Chirizzi, Daniela
Chiu, Nan-Fu
Chiu, Tai-Chia
Cho, Gihwan
Cho, Jae Yoong
Cho, Jin-ho
Cho, Jung Hwan
Cho, Kyungeun
Cho, Sung Ho
Cho, William
Cho, Young Im
Choi, Hojong
Choi, Inhee Curie
Choi, Jae-Won
Chon, Ki H.
Chong, Zan-Kai
Choo, Hyunseung
Choo, Jaebum
Chou, Chun Tung
Chou, Jung-Chuan
Chou, Kan-Sen
Chow, Jacky
Chow, Tommy Wai-Shing
Chowhdury, Sazzadur
Choy, Yat-Sze
Chronis, Nikos
Chrostowski, Lukas
Chu, Fulei
Chu, Jinkui
Chu, Shu-Chuan
Chu, Tianxing
Chuang, Cheng-hung
Chuang, Cheng-Long
Chuang, Po-Jen
Chui, Hsiang-Chen
Chun, Honggu
Chung, Ching-Che
Chung, Deborah
Chung, Jae-Hyun
Chung, Sun-Ok
Ciaccheri, Leonardo
Ciampa, Francesco
Ciecierega, Thomas
Cigna, Francesca
Cikajlo, Imre
Ciminelli, Caterina
Cipresso, Pietro
Cipriano, Aldo
Ciurana, Joaquim
Ciuti, Gastone
Clark, Heather A.
Clarke, Peter J.
Clausen, Ingelin
Claussen, Jonathan C.
Cleland, Ian
Cleverly, James
Cliffel, David E.
Clinton, Smith
Clivati, Cecilia
Close, Dan
Cocconcelli, Marco
Cochran, Sandy
Cochrane, Cédric
Cocolios, Thomas Elias
Coelho, João M. P.
Coelho, João Pinto
Colbert, Christopher L.
Colitti, Walter
Collier, Rem
Collin, Jussi
Collins, John
Collins, Scott D.
Collins, Stephen
Collotta, Mario
Colot, Olivier
Coltekin, Arzu
Concina, Isabella
Coninx, Kristof
Constandinou, Timothy
Contin, Alfredo
Contreras, Pedro
Contreras-Medina, Luis
Contreras-Vidal, Jose Luis
Corato, Francesco Di
Corchado, Juan M.
Corcoran, Peter
Cordero Fuertes, Juan Antonio
Corigliano, Alberto
Coronato, Antonio
Correa, Christian
Cortes, Pedro
Corujo, Daniel
Coscetta, Agnese
Cosnier, Serge
Cossairt, Oliver (Ollie)
Costa Branco, P.J.
Costa, Ângelo
Costa, Daniel
Costa-García, Agustín
Cotos, José M.
Cottone, Francesco
Courtney, Charles
Covington, James
Cozzolino, Daniel
Cragg, Peter J.
Cramer, Michael
Crew, Adrian
Crick, Christopher
Cristina, Ana Majarena
Croce Jr., Robert A.
Crowcroft, Jon
Cruy Silva, Rodolfo
Cruz, Jesus Manuel de la
Cui, Daxiang
Cui, Jiangfeng
Cui, Lina
Culshaw, Brian
Cummins, Gerard
Cusano, Andrea
Cutti, Andrfea Giovanni
Cvetkovski, Goga
Czajkowski, Jakub
D’Lima, Darryl D.
Da Silvab, Joaquim C.G. Esteves
Dahlin, Andreas
Dai, Hongjun
Dai, Nan
Dai, Rui
Dai, Yuchao
Dai, Zong
Daigle, Courtney
Dale, Laura M.
Daliri, Ali
Dalla Chiara, Bruno
Danescu, Radu Gabriel
Daniele, Passeri
Danson, Mark
Dao, Tien Tuan
Dasgupta, Prithviraj
Dattelbaum, Jonathan D.
David, Spierer
Davidsson, Paul
Davie, Andrew
Davies, Gareth
Davitt, Kristina
Dawoud, Amer
Dawson, Martin
de Adana, Francisco Saez
de Boer, Steven F.
de Bruin, Sytze
de Cesare, Fabrizio
de Cola, Tomaso
de Gennaro, Gianluigi
de la Hoz Siegler, Hector
de la Rosa, Ramon
de Lima, Jr., Mauricio M.
de Lope, Javier
de Maeztu, Leonardo
de Marcellis, Andrea
de Paz Santana, Juan Francisco
de Pietro, Giuseppe
de Rossi, Stefano
de Souza Filho, Carlos
de Souza, Paulo Antonio
Deambrogio, Lina
Decotignie, Jean-Dominique
Dehzangi, Omid
Deiab, Ibrahim
Del Din, Silvia
Del valle, Manel del Valle
Del Ventisette, Chiara
Delalieux, Stephanie
Delashmit, Walter
Delmas, Jean Pierre
Delnavaz, Aidin
Delsing, Jerker
Delvigne, Frank
Demiris, George
Deng, Daniel
Deng, Ming
Deng, Mingxi
Deng, Zhihong
Dennis, Allison
Deravi, Leila F.
Deserno, Thomas M.
Dettmar, Uwe
Detweiler, Carrick
Devitt, Dale
di Barba, Paolo
di Pasquale, Fabrizio
di Stefano, Giuseppe
Diamant, Roee
Dias, Jorge
Dias, Nuno
Díaz Blanco, Manuel Jesús
Díaz-Cruz, José Manuel
Díaz-Vilariño, L.
Dickert, Franz L.
Dickey, Michael D.
Dickson, Wayne
Didar, Tohid Fatanat
Dieber, Bernhard
Diller, Tom
Ding, Bing
Ding, Jow-Lian
Ding, Ming
Ding, Yong
Dios, Jr. Martinez-de
Diraco, Giovanni
Diwakar, Prasoon
Dixon, Steve
Djendi, Mohamed
Djerdir, Abdesslem
Djurić-Jovičić, Millica
Djurović, Igor
Dmitriev, Ruslan
Do, Yongtae
Dobre, Ciprian
Dogancay, Kutluyil
Dogramadzi, Sanja
Doh, Nakju
D’Oleire-Oltmanns, Sebastian
Doll, Joey
Domínguez Renedo, Olga
Donelli, Massimo
Dong, Guangming
Dong, Junhang
Dong, Lei
Dong, Lili
Dong, Mingsheng
Dong, Tina T. X.
Dong, Xiuzhu
Dong, Ying
Dong, Ze
Dong-Hwan, Hwang
Donzella, Valentina
D’Orazio, Tiziana
Dorsey, Kristen L.
Douligeris, Christos
Dovis, Fabio
Drexler, Petr
Drobics, Mario
Drost, Bertram
Du Burck, Frederic
Du, K.-L.
Du, Ke
Du, Zhengchun
DUAN, Fabing
Duan, Fajie
Duan, Haibin
Duan, Lian
Duan, Zhansheng
Duann, Jeng-Ren (JR)
Dubey, Venky
Dubovyk, Olena
Ducey, Mark J.
Duda, Krzysztof
DULSKI, Rafał
Dunbar, Alan
Dunne, Thomas
Duran-Cantolla, Joaquin
Durfee, William
Duroc, Y.
Dussy, Stephane
Dvinskikh, Sergey
Dyke, Shirley
Dzamba, Tom
Dzantiev, Boris
Dziuda, Łukasz
Eaton, Mark Jonathan
Ebrahimi, Dariush
Echeverría, Jesús
Eckert, Sven
Eduard, Llobet
Edwan, Ezzaldeen
Edwards, Matthew E.
Edwards, Rm
Eftekhar, Amir
Eichholz, Jörg
Eichstädt, Sascha
Eizenman, Moshe
Ekstedt, Mathias
Ektesabi, Mehran Motamed
El Saddik, Abdulmotaleb
ElBatanouny, Mohamed
Elberink, Sander Oude
Eldén, Lars
Elefsiniotis, Alexandros
Elena, De Momi
Eliasson, Jens
Elmogy, Ahmed
El-Osery, Aly I.
Elphinstone, J. G.
El-Sayed, Amr M.
Emadi, Tahereh Arezoo
Emokpae, Lloyd
Endo, Tatsuro
Engelen, Peter-Jan
Enright, John
Ercan, M Fikret
Erhart, Jiri
Ersoy, Cem
Esposito, Fabio
Estrela, Pedro
Everaerts, Jurgen
Evrendilek, Fatih
Evtugyn, Gennady
Ewart, P.
Fabbrocino, Giovanni
Fabregas, Ernesto
Fabris, Laura
Faburay, Bonto
Faezipour, Miad
Fagan, Colett
Falcon, Rafael
Falcone, Francisco
Falk, Tiago H.
Falkner, Nick
Falletta, Ermelinda
Fally, Martin
Falzon, Greg
Fan, Chunhai
Fan, Guoliang
Fan, Li
FAN, Pingyi
Fan, Xinxin
Fan, Xudong
Fang, Hongliang
Fang, Jiancheng
Fang, Peng
Fang, Shih-Hau
Fang, Xiaosheng
Fang, Ye
Fang, Yuming
Farag, Fawzy F
Faria, Dalva Lúcia Araújo de
Farinella, Giovanni Maria
Fasano, Giancarmine
Fathallah, Fadi
FAURE, Denis
Favero-Longo, Sergiole.
Fearn, Tom
Fechteler, Philipp
Fei, Gaolei
Fei, Juntao
Feichtinger, Hans Georg
Feiertag, Gregor
Feilberg, Anders
Felisberto, Paulo
Feng, Yunxia
Feng, Zhi Hua
Feng, Zhipeng
Fensel, Anna
Fensli, Rune
Fernández Caballero, Antonio
Fernández, Alberto
Fernández, Antonio
Fernandez, Eduardo
Fernández, Eduardo
Fernández, Luis Ángel
Fernandez, Luis J.
Fernández, Roemi
Fernández-Abedul, M. Teresa
Fernandez-Llatas, Carlos
Fernández-Sánchez, César
Ferraro, Pietro
Ferré-Borrull, Josep
Ferreira, Elnatan
Ferreira, Luis Lino
Ferri, Giuseppe
Ferrigno, Luigi
Ferroni, Matteo
Fiaidhi, Jinan
Filip, Jiri
Filipe Costa, Manuel
Filipe, Doutor Vitor Manuel Jesus
Filippi, Anthony
Filomeno Carlos Viegas, Domingos Xavier
Fingas, Merv
Fiondella, Lance
Fiorani, Luca
Fiorini, Carlo
Fischer, Andreas
Fite, Kevin
Fitz-Gerald, James M.
Flammini, Francesco
Flater, Erin E.
Flechsig, Gerd-Uwe
Flemmer, Claire
Fletcher, Jacqueline
Fletcher, Mary
Fleury, Bernard Henri
Flokstra, Jaap
Flórez-Revuelta, Francisco
Florsch, Nicolas
Foh, Chuan Heng
Fonollosa, Jordi
Fonseca, Viviane Muniz
Fontaine, Jean-françois
Fontana, Marco
Foo, Ernest
Ford, Jason
Foresti, Gl
Fornaciari, Michele
Förster, Anna
Forsythe, Stephen
Fortuna, Luigi
Foschini, Luca
Foster, Robert
Foster, Scott
Foti, Dora
Fotiadis, Dimitrios
Fox, Glen
Fraga, M. A.
Franco, Annalisa
Francomano, Maria Teresa
Frank, Hedrich
Fred, Ana
Freymueller, Jeff
Frischholz, Robert
Fröhlich, Antônio Augusto
Fruk, Ljiljana
Fu, Yue Jun
Fu, Huirong
Fu, Jun
Fu, Liling
Fu, Weiling
Fuh, Yiin-Kuen
Fujii, Mahito
Fujii, Yoshiaki
Fujimoto, Takaaki
Fujita, Katsumasa
Fujiura, Tateshi
Furferi, Rocco
Furth, Paul M.
Fusiek, G.
Gabriele, Cambiotti
Gachet Páez, Diego
Gagliardi, Gianluca
Gaglio, Salvatore
Galán-Mascarós, José Ramón
Galán-Vidal, Carlos
Galiatsatos, Niko
Galip Saracoglu, Ömer
Galipeau, David W
Gall, Juergen
Galli, Iacopo
Gallo, Orazio
Galstyan, Vardan
Gambi, Ennio
Gamo, Javier
Gams, Matjaž
Gan, Ning
Ganapathy, Senthil
Gandino, Filippo
Gansterer, Wilfried N.
Gao, Chenqiang
Gao, Ding
Gao, Huijun
Gao, Junqi
Gao, Lu
Gao, Peng
Gao, Shesheng
Gao, Weilu
Gao, Xiaofeng
Garbay, Catherine
García García, María Soledad
García Zapirain, Begoña
García, Carmelo
Garcia, Cecilia
García, Fernando
García-Diego, Fernando-Juan
García-Hernando, Ana-Belén
Garcia-Sanchez, Antonio-Javier
Garcia-Sanchez, Felipe
García-Sánchez, P.
Garcia-Valls, Marisol
García-Zubia, Javier
Gargano, M.
Gargiulo, Gaetano D
Garozzo, Domenico
Garratt, Matt
Garrett, David J
Garrido-Varo, Ana
Garulli, Andrea
Gasparini, Leonardo
Gasparrini, Samuele
Gasteratos, Antonios
Gates, Byron D
Gauglitz, Guenter
Ge, Fengxiang
Ge, Maorong
Ge, Xudong
Ge, Yunjian
Geil, Mark
Geladi, Paul
Gelembe, Erol
Gemeiner, Peter
Genderen, Piet van
Geng, Jianghui
Georgakis, Apostolos
Georgantas, Nikolaos
Georgieva, Elena
Georgoulas, Christos
Georgoulas, George
Ghayesh, Mergen H.
Ghio, Alessandro
Giacomozzi, Claudia
Gianfrani, L.
Gibson, Christopher T.
Gibson, Laurie
Gidlund, Mikael
Gierak, Jaques
Giese, Holger
Gikas, Vassilis
Gil, Arturo Aparicio
Gil, Eduardo Pinilla
Gil, Emilio
Gil, Pablo
Gingl, Zoltan
Gini, Giuseppina
Giorgetti, Andrea
Giorgi, Gabriele
Giouroudi, Ioanna
Girão, Pedro S.
Girousi, Stella
Giubileo, Gianfranco
Glas, Dylan F.
Glavin, Martin
Gleason, Scott
Glennie, Craig
Gloaguen, Richard
Gnecchi, J.A. Gutiérrez
Godfrey, Alan
Goeman, Werner
Goethals, Pauwel
Gokaraju, Balakrishna
Golatowski, Frank
Goldthorpe, Irene
Golemati, Spyreta
Goljan, Miroslav
Gollmann, Dieter
Goltz, Douglas
Golzarian, Mahmood
Gomes, Danielo
Gomez Gutiérrez, A.
Gómez Mancilla, Julio César
Gomez-Gil, Jaime
Gonçalves, Luiz M. G.
Gonçalves, M. Sameiro T.
Gong, Jiaqi (Jackey)
Gong, Myoung-Seon
Gong, Peng
Gong, Qihuang
Gong, Xiaojin
Gong, Zheng (Roger)
González Jorge, Higinio
Gonzalez Ruiz, Vicente
González, Apolinar
Gonzalez, Jesus
Gonzalez, Joaquin
Gonzalez, Miguel
González-Abril, Luis
Gorricho, Juan-Luis
Goryacheva, Irina Yu.
Goss, Larry
Gosselin, Benoit
Gotsis, Konstantinos
Gouvêa, Paula M. P.
Gracias, David
Grammalidis, Nikos
Grandner, Michael A.
Granitzer, Petra
Grant, James
Grau, Antoni
Gravenkamp, Hauke
Gravina, Raffaele
Gray, Brian
Greenbaum, Alon
Greene, Eric C.
Gregory, John
Grenzdoerffer, Goerres
Greswell, Ricahrd
Grönvall, Erik
Groom, Geoff
Gross, Richard
Große, S
Groves, Kathy
Gruber, Jonas
Gruen, Armin
Gruev, Viktor
Gu, Fengshou
Gu, Jason
Gu, Qijun
Gu, Qingyi
Gu, Yu
Guan, Haiyan
Guan, Quansheng
Guan, Yan
Guan, Zhangyu
Gubbi, Jayavardhana
Guedes, Luiz
GÜEMES, ALFREDO
Guerrero, Cesar
Guerriero, Francesca
Guette, Gilles
Gui, Guan
Guidetti, Riccardo
Guidi, Francesco
Guitton, Alexandre
Guivant, Jose
Guldiken, Rasim
Guo, Guodong
Guo, Tuan
Guo, Ying
Guo, Zhiqian
Gupta, Mool C.
Gurkan, Umut Atakan
Gutierrez, Agustín
Gutiérrez, Daniel
Gutierrez, Diego
Gutiérrez, Juan Manuel
Gutiérrez, S.
Gutierrez-Blanco, Oscar
Guy, Chris
Haber-Pohlmeier, Sabina
Habib, Ayman F.
Hadid, Abdenour
Haick, Hossam
Haider, Mohammad Rafiqul
Hale, J M
Hall, Adam
Hall, Charles B.
Hall, David
Haller, Merrick
Hallsworth, J.E.
Hambly, Adam
Hamid, Nafiz Imtiaz Bin
Hamilton, Robert G.
Hamilton, Tara
Hämmerle, Martin
Hammerschmidt, Kurt
Han, B.G.
Han, Dong Yeob
Han, Jen-Yu
Han, Jiaguang
Han, Joon Hee
Han, Jungong
Han, Ke
Han, Min
Han, Ming
Han, Richard
Han, Song
Handyside, Cameron
Hank, Tobias Benedikt
Hanke, Klaus
Hanlen, Leif
Hanna, Radecka
Hannam, Kirsty E.
Hansen, John H. L.
Hanson, Kenneth
Hanson, Mark
Hanson, Ronald K.
Harazim, Stefan M.
Harima, Ken
Harland, Robert Carl
Harle, Robert
Hart, John P.
Hart, Ronald
Hartmann, Anton
Hasar, U.C.
Hashiguchi, Gen
Hassan, Aseel
Hassan, Mohammad Mehedi
Hatzinakos, Dimitrios
Hauptmann, Peter
Havlík, Jan
Havran, Vlastimil
Hawrylak, Peter J.
Hayashi, Kenshi
Hayashibe, Mitsuhiro
Hazrati, Mehrnaz
He, Bin
He, Bo
He, Cunfu
He, David
He, Jianping
He, Liang-Hua
He, Lina
He, Qingbo
He, Ran
He, Xiuli
He, Yawen
He, Yuhong
He, Zhenwen
Healy, William M.
Hebel, Martin A.
Heidrich, Wolfgang
Heijden, R.W. (Rob) van der
Heiskanen, Arto
Heitzinger, Clemens
Helms, John
Helten, Thomas
Hempel, Michael
Henebry, Geoffrey M.
Henesey, Lawrence E.
Heng, Sabrina
Henriques, J.
Heo, Junyoung
Hepel, Maria
Her, Shiuh-Chuan
Herberg, Ulrich
Herbert, John
Herda, Trent J.
Hernández, Luis
Hernández-Andrés, Javier
Herrera, E. Pulido
Herrera, Jordi
Herrera-May, A. L.
Hervás Martínez, César
Herwig, Christoph
Herzog, Grégoire
Heuer, Henning
Heunecke, Otto
Hianik, Tibor
Higuchi, Takaya
Hijji, Yousef
Hill, Andrew
Hill, Daniel
Hill, Kelly
Hinders, Mark
Hintermueller, Christoph
Hirose, Kiyoshi
Hirsch, Thomas
Hirt, Christian
Hladky-Hennion, Anne Christine
Hlawatsch, Franz
Hnat, William P.
Ho, Chao-ching
Ho, Hopui
Ho, Johnny Chung Yin
Hochberg, Eric
Hodneland, Erlend
Hoffmeister, Dirk
Hogsette, Jerome A.
Hölbl, Marko
Holgado, Juan A.
Holmberg, Hans Christer
Holmes, Christopher
Holownicki, Richard
Holroyd, Michael
Holzer, Clemens
Holzinger, Andreas
Honda, Shinya
Honeine, Paul
Hong, Jiman
Hong, Keum-Shik
Hong, Liang
Hong, Men
Hong, Min-Cheol
Hong, Xiaopeng
Hong, Xin
Hongliang, Ren
Hoorfar, Mina
Horeman, Tim
Hormati, Ali
Hornak, Joseph P.
Horng, Mong-Fong
Horrillo, María del Carmen
Horvath, György
Hossain, A.K.M. Azad
Hossain, A.K.M. Mahtab
Hossain, Alamgir
Hou, Liqun
Hoxley, David
Hsieh, Chih-Cheng
Hsieh, Hung-Yun
Hsieh, Jun-Wei
Hsin, Yue-Ming
Hsu, Chien-Chang
Hsu, Chien-Lung
Hsu, Chih-Shun
Hsu, Chun-Fei
Hsu, Hui-Huang
Hsu, Jui-Ming
Hsu, Miao-Ju
Hsu, Yeh-Liang
Hu, Chao
Hu, Fei
Hu, Fengqing
Hu, Jia
Hu, Jingtong
Hu, Ning
Hu, Zhu
Hu, Sanqing
Hu, Shuguo
Hu, Shuo-Cheng
Hu, WeiHua
Hu, Xiaogang
Hu, Xiaogong
Hu, Yong
Hu, Yuh-Chung
Hu, Zhebin
Hua, Hui Guo
Huang, Chih-Chung
Huang, Dong
Huang, Gong-Sheng
Huang, Guangbin
Huang, Hong
Huang, I-Yu
Huang, Jhin-Fang
Huang, Jingyun
Huang, Kuan-Wen
Huang, Lei
Huang, Liquan
Huang, Ming-Chun
Huang, Qian
Huang, Qing-An
Huang, Qinghua
Huang, Tiao-Yuan
Huang, Tien-Chi
Huang, Wei
Huang, Wei Min
Huang, Wei-Hua
Huang, Wenbin
Huang, Xiaopeng
Huang, Xin
Huang, Xingfeng
Huang, Xing-Jiu
Huang, Xinming
Huang, Yanbo
Huang, Ying
Huang, Yo-Ping
Huang, Yueh-Min
Huang, Zhaorong
Huang, Zi-Gui
Huaqing, Wang
Huber, Alexander
Hudak, Andrew
Hughes, Michael P.
Huguet, Silvia Soria
Huh, Eui Nam
Huh, You
Hung, H.L.
Hung, Jui-Chung
Hung, Min-Hsiung
Hunsche, Mauricio
Hunt, Heather
Hur, Jin
Hurtado, María José
Hussein, Mohamed
Hwang, Han-Jeong
Hwang, Hoyoung
Hwang, Jenq-Neng
Hwang, Min-Shiang
Hwang, Phil
Hwang, Suk-Seung
Hwang, Yongha
Hwang, Yoola
Hwang, Yuan-Chu
Hyyppä, Juha
IBUKI, Tatsuya
Iera, Antonio
Iervolino, Elina
Iglesias, José Antonio
Iglesias, Roberto
Igreja, Rui
Igual, Raul
Iiames, John Shepherd
Iida, Michihisa
Ijima, Hiroyuki
Ikehara, T
Ikonen, Erkki
Iljaž, Rade J
Incel, Ozlem Durmaz
Infantino, Alessandro
Ingalls, Todd
Ingebrandt, Sven
Ino, Kosuke
Iocchi, Luca
Ioppolo, Tindaro
Iosa, Marco
Isacsson, A.
Islam, Syed Kamrul
Itkis, Mikhail E.
Itoh, Tetsuji
Ivask, Angela
Iwata, Tetsuo
Iyota, Taketoshi
Jaballah, Wafa Ben
Jacobs, Peter
Jacobson, M.D.
Jacopo, Aguzzi
Jaffrezic-Renault, Nicole
Jafra, Sylwia
Jaganathan, Arun
Jaime, Castillo-León
Jakubik, Wiesław P.
Jamali, Mohsin
Jamali, Mohsin M.
James, Daniel
Jamshidi, Mohammad
Jan, Shau-Shiun
Jan, Skaloud
Jang, Gil-Jin
Jang, Liping
Jannett, Thomas C.
Jastrzebski, Rafal
Javanmard, Mehdi
Javidi, Bahram
Javier, Francisco Otamendi
Jaw, Jen-Jer
Jaworowski, Cheryl
Jayaraman, Prem Prakash
Jayaraman, Sundaresan
Jeffrey D., Brewster
Jeng, Jen-Tzong
Jenkins, Daniel M.
Jensen, Jennifer
Jentsch, Florian
Jeon, Doyoung
Jeong, Gu-min
Jeong, Hae-Duck Joshua
Jérémy, Butet
Jerschow, Alexej
Jhe, Wonho
Ji, Hongli
Ji, Kang Hyeun
Ji, Shouling
Jia, Xinhua
Jiang, Brendan
Jiang, hongbo
Jiang, Joe-Air
Jiang, Kaili
Jiang, Ning
Jiang, Peng
Jiang, Ping
Jiang, Ruinian
Jiang, Xiaoguang
Jiang, Xiaohong
Jiang, Xiaoning
Jiang, Xin
Jiang, Xuchuan
Jiang, Yifei
Jiang, Yuqiang
Jimenez, Cecilia
Jimenez, Felipe
Jiménez, Víctor P. Gil
Jiménez-Muñoz, Juan C
Jin, Guoyong
Jin, Qinghui
Jin, Ren Cheng
Jin, Shuanggen
Jin, Xiaojun
Jin, Xin
Jin, Zhanpeng
Jirka, Simon
Joerger, Mathieu
Johanson, Urmas
Johansson, Tim
John, Dinesh
Johnson, Mark
Johnson, Mark S.
Johnson, Nigel P.
Johnson, Thomas
Jones, Barry
Jones, John D.
Jones, Scott B.
Jones, Steven
Jongschaap, Raymond
Jorge, Pedro
José, Rui
Josiński, Henryk
Jovanov, Emil
Juang, Jyh-Ching
Judith, Rishpon
Jung, Andras
Jung, Byung Jun
Jung, Hyunyoung
Jung, Jin-Woo
Jung, Low Tang
Jung, Markus
Jung, Sang-Joong
Jung, Seong-Ook
Jung, Seul
Jung, Soo-Yong
Juričić, Đani
Jurij, Rakun
Jwo, Dah-Jing
Kaburlassos, Vassilis G.
Kadone, Hideki
Kaftandjian, Valérie
Kahrizi, Mojtaba
Kaiser, Jozef
Kaivosoja, Jere
Kalaji, Hazem M.
Kalantar, Kourosh
Kalcher, Kurt
Kaler, Karan
Kalivas, Grigorios
Kalogiros, John
Kaltsas, Grigoris
Kamsu-Foguem, Bernard
Kan, Yao-Chiang
Kandris, Dionisis
Kang, Bumjoon
Kang, Byeong Ho
Kang, Byong-Ho
Kang, Chulhun
Kang, Dong-Joong
Kang, Hangbong
Kang, Soon Ju
Kang, Zhizhong
Kannan, Rajgopal
Kanno, Ariyo
Kanoun, Olfa
Kanta, Bera Lakshmi
Karanassios, Vassili
Karapanagiotis, Ionnis
Karczmarek, Paweł
Karimi, Hassan A.
Karlgaard, Christopher David
Karstoft, Henrik
Kartsakli, Elli
Karuei, Idin
Karvonen, Heikki
Kasneci, Enkelejda
Kastner, Ryan
Katapodis, Petros
Kato, Fumitake
Katsis, C.D.
Katupitiya, Jayantha
Katz, Randy
Kaushik, Ajeet
Kavanagh Be, Richard
Kawakami, Jun
Kawarabayashi, Jun
Kawasaki, Haruhisa
Kažys, Rymantas
Keating, Adrian
Kedzierski, Michal
Kee, Changdon
Kefayati, Gholamreza H.R.
Kehagias, Athanasios
Kemp, John
Kemper, Björn
Kempers, Roger
Kennel, Ralph
Kerman, Kagan
Keshavan, Jishnu
Ketterling, Jeffrey A.
Khaleghi, Bahador
Khan, Adil Mehmood
Khan, Jamil Y.
Khan, Mohidus Samad
Khan, Shfaqat Abbas
Khan, Usman
Khan, Usman A.
Khan, Zaheer
Khan, Zahoor
Khati, Makobetsa
Khushaba, Rami
Kibriya, Muhammad G.
Kiefer, Peter
Kilroy, Joseph P.
Kim, Ayoung
Kim, Bok-Hyun
Kim, Dae-Hyeong
Kim, Dong-Eun
Kim, Euntai
Kim, Ghiseok
Kim, Hong-Seok
Kim, Howon
Kim, Hwan Myung
Kim, Hyejung
Kim, Hyun-Sung
Kim, J.-H.
Kim, Jaehwan
Kim, Jong Seung
Kim, Jong-Hoon
Kim, Jung Tae
Kim, Ki-Hyung
Kim, Kwang S.
Kim, Kyoungsik
Kim, Min Young
Kim, Mooyoung
Kim, Sang-Jae
Kim, Seok-Min
Kim, Seon Joo
Kim, Sin
Kim, Siyong
Kim, Sohee
Kim, Sungho
Kim, Sunwook
Kim, Whoi-Yul
Kim, Wonjun
Kim, Yoon-Chul
Kim, Young-Kee
Kim, Yousok
Kimura, Nobutada
Kinet, Damien
King, Chung-Ta
Kinkeldei, Thomas
Kinnunen, Teemu
Kintzios, Spiridon
Kiourti, Asimina
Kiranoudis, Chris T.
Kirchgessner, Norbert
Kirsanov, Dmitry
Kivistö, Rauni
Kizek, Rene
Klatzky, Roberta
Klausmeyer, Philip
Kleih, Sonja
Klessig, Henrik
Klumperink, Eric
Knapp, Karen M.
Kneip, Laurent
Knief, Peter
Knobbe, Arno
Knopp, Dietmar
Ko, ByoungChul
Ko, Chia-Nan
Ko, Hoon
Ko, Hyoungho
Ko, Nak Yong
Ko, Ren-Song
Ko, Wen H.
kocbach, Jan
Kociuba, Waldemar
Kodera, Noriyuki
Koenig, Alexander
Koh, Jaehan
Kohoutek, Tobias
Kokkinos, Panagiotis
Kolasa, Marta
Kolb, Armin
Kolehmainen, Mikko
Kolla, Reiner
Kolomvatsos, Kostas
Kolozali, Sefki
Koltunowicz, Tomasz
Koncar, Vladan
Kong, Wanzeng
Kono, Miyuki
Konry, Tania
Konstantopoulos, Charalampos
Konttinen, Yrjö T.
Kopacek, Peter
Korampally, Madhuri
Korampally, Venumadhav
Korpeoglu, Ibrahim
Koser, Kevin
Kosmas, Panagiotis
Kosmopoulos, Dimitrios
Kosterink, Stephanie
Kotropoulos, Constantine
Kotsifakos, Alexios
Kotti, Margarita
Kotulski, Zbigniew
Kouchi, Makiko
Koukoulas, Triantafillos
Koulamas, Christos
Koumoulis, Dimitrios
Kouroupetroglou, Georgios
Kouroussis, Georges
Koutsouris, Dimitrios
Kovačič, Stanislav
Koyama, Daisuke
Koziorek, Jiri
Kozlov, Alexander G.
Krawczuk, M.
Krejcar, Ondrej
Krief, Francine
Kristensson, Per Ola
Kroeger, Silke
Królicka, Agnieszka
Kronberger, Rainer
Kroschel, Kristian
Krüger, Björn
Ksiezopolski, Bogdan
Kuang, K. S. C.
Kuang, Kevin
Kuhlmann, Heiner
Kulathumani, Vinod
Kumar, Suman
Kuo, Chil-Chyuan
Kuo, Chung-Hsien
Kuo, Chung-Yen
Kuo, Ying-Che
Kuochih, Chuang
Kurbatov, A.M.
Kurillo, Gregorij
Kurkuri, Mahaveer
Kusakunniran, Worapan
Kusyk, Janusz
Kuusniemi, Heidi
Kwak, Kyung Sup
Kwak, Moon K.
Kwong, Chan Pak
Kwong, Wing C.
La, H.M.
La, Hung Manh
Labeed, Fatima H
Lachapelle, Gérard
Lachman, Noa
Ladikos, Alexander
Laefer, Debra F.
Laffort, Paul
Lafratta, Christopher
Lagkas, Thomas
Lagüela, S.
Lai, Chao-Sung
Lai, Jianhuang
Lai, Stefano
Lakakis, Konstantinos
Lakard, Boris
Lallart, Mickael
Lam, Toby H.W.
Lam, Wilbur A.
Lampoltshammer, Thomas J.
Lan, Minbo
Lanceros-Mendez, Senen
Laney, Sam
Lang, Walter
Lang, Yin H.
Lanzola, Giordano
Lapira, Edzel R.
Larcher, Luca
Largillier, Thomas
Larin, Kirill
Larios, Diego F.
Lau, Lawrence
Lau, Rynson W. H.
Lauer, Martin
Laurin, Gaia Vaglio
Lausch, Angela
Lavagno, Luciano
Lay, Marcus D.
Lay-Ekuakille, Aimé
Lazarescu, Mihai T
Lazaro, Antonio
Le Bars, Fabrice
Le Pioufle, Bruno
Le, Lawrence H.
Le, Qiang
Leach, Richard
Leahy, Kevin
Leardi, Riccardo
Leardini, Alberto
Leccese, Fabio
Lecuire, Vincent
Lee, Boreom
Lee, Byeong Ha
Lee, Byoungho
Lee, Chang-Gun
Lee, Chao-Hsien
Lee, Cheng-Chi
Lee, Chia-Yen
Lee, Chien-Nan
Lee, Chi-Yuan
Lee, Chongmu
Lee, David
Lee, Dr Learn Han
Lee, Heung-No
Lee, Hsiang-Chieh
Lee, Hsien-Ming
Lee, Hyung-Kun
Lee, Hyunjoo J.
Lee, In-Kwon
Lee, Jae Sung
Lee, Jay
Lee, Jong-Heun
Lee, Jung Keun
Lee, Ju-Yi
Lee, Kiryung
LEE, Kong-Aik
Lee, Min Hyung
Lee, Moon Ho
Lee, MunKyu
Lee, Sang-Gon
Lee, Sang-Heon
Lee, Sangyoun
Lee, Seung-Yop
Lee, Shie-Jue
Lee, Suk Jin
Lee, W M Steve
Lee, Woo-Kyun
Lee, Wooyoung
Lee, Yong Wook
Lee, Young-Koo
Lefèvre, Eric
Legland, David
LEHSAINI, Mohamed
Lei, Chi-Un
Lei, Hong
Lei, Kang
Lei, Yaguo
Leis, John
Leitner, Gerhard
Leivseth, Gunnar
Leng, Yong-Gang
Lenzen, Christoph
Leone, Alessandro
Leone, Pierre
Lepert, Guillaume
Leppänen, Teemu
Lerner, Amit
Lerosey, Geoffroy
Lesiak, Piotr
Leu, Tzong-Shyng
Leung, Yiu-wing
Levassort, Franck
Levesque, Martin
Levin, Robert E.
Levy, Cesar
Lewin, Peter A.
Leygraf, Christofer
Li, Baoxin
Li, Bin
Li, Changle
Li, Chang-Tsun
Li, Changzhi
Li, Chengqing
Li, Chun-Ta
Li, David
Li, Fei
Li, Fengyuan
Li, Gang
Li, Guo-Dong
Li, Guoqiang
Li, Guozheng
Li, Haiyun
Li, Hao
Li, Jiaming
Li, Jian
Li, Jian-Wei
LI, Jian-Xun
Li, Jinbao
Li, Jingsong
Li, Jun
Li, Junhong
Li, Kwok Hung
Li, Lin
Li, Na
Li, Peijun
Li, Peiwu
Li, Qian
Li, Qiao
Li, Qing
Li, Qingquan
Li, Qipeng
Li, Qiuhong
Li, Qun
Li, Shancang
Li, Shang
Li, Shuai
Li, Weihua
Li, Wen
Li, Xiang
Li, Xiaoli
Li, Xiaolong
Li, Xiaopeng
Li, Xiaoyuan
Li, Xiu-Zhi
Li, Yang
Li, Yi
Li, Yong
Li, Zhaoliang
Li, Zhao-Liang
Li, Zheng-Zhou
Li, Zhi
Li, Zhuo
Lian, Feng-Li
Liang, Chiu-Kuo
Liang, Jun
Liang, Xiaohul
Liang, Xuefeng
Liang, Yu
Liang, Zhitao
Liao, Bin-Yih
Liao, Lei
Liao, Lun-De
Liao, Qi
Liao, Shengcai
Liao, Wen-Jiao
Liao, Xiaofei
Liao, Yi-Hung
Lieberzeit, Peter
Lien, Shao-Yu
Lien, Yao-Nan
Lim, Hyuk
Lim, M. K.
Lim, Seokbin
Lim, Sungkyun
Lin, Bor-Shyh
Lin, Chi
Lin, Chia-Chen
Lin, Chih-Ming
Lin, Chii-Wann
Lin, Chin-Feng
Lin, Fanglue
Lin, Fuhua
Lin, Hui
Lin, Hung-Yin
Lin, Jium-Ming
Lin, Jr-Lung
Lin, Kuo Chi
Lin, Kwan-Hwa
Lin, Liwen
Lin, Ming-Bo
Lin, Pei-Chun
Lin, Qiang
Lin, S.T.
Lin, Sebastian
Lin, Sidney
Lin, Tao
Lin, Ting-Lan
Lin, Wei
Lin, Wei-Yang
Lin, Xiangguo
Lin, Xu
Lin, YC
Lindell, Michael
Lindgren, Helena
Lindsey, Brooks D
Linnamo, Vesa
Linnhoff-Popien, Claudia
Liter, Siek
Little, Jim
Liu, Aiqun
Liu, Bin
Liu, Changjun
Liu, Chengbin
Liu, Chung Chiun
Liu, Dalong
Liu, Dehong
Liu, Harold
Liu, Hongwei
Liu, J. Z
Liu, Jindong
Liu, Jing
Liu, Jingbin
Liu, Jinhai
Liu, Jinyun
Liu, Junfeng
Liu, K
Liu, Kebin
Liu, Ming
Liu, Peixiang
Liu, Qiong
Liu, Shing-Hong
Liu, Shouyin
Liu, Tao
Liu, Wei-Cheng
Liu, Wen Chung
Liu, Xin
Liu, Xinhui
Liu, Xinxin
Liu, Xinyu
Liu, Xiuhui
Liu, Xuan
Liu, Yang
LIU, Yanjun
Liu, Yi
Liu, Yingying
Liu, Yonggang
Liu, Yuling
Liu, Yunhuai
Liu, Zheng
Liu, Zhengjun
Liu, Zhong
Liu, Ziping
Liz Crespo, María
Llorens Calvedas, Jordi
LloydSpetz, Anita
Lo Presti, Letizia
Lo, Chien-Fong
Lo, Yu-Lung
Lobo, Felipe
Lobo, Jorge
Locatelli, Clinio
Loccufier, Mia
Lochman, Steffen
Logsdon, S. D.
Loh, Kenneth
Lohan, Elena Simona
Loidl, Martin
Loke, Seng W.
Lomer, Mauro
Lonardo, Pietro
Long, Feng
Longa, Patrick
Longo, Domenico
Lonini, Luca
Looney, David
Lopes, António M.
Lopes, Waslon Terllizzie Araujo
López, Antonio
López, Antonio M.
Lopez, Joaquín
Lopez, Juan
Lopez, Larry
Lopez, Tomas Sanchez
López-Cózar, Ramón
López-de-Ipiña, Diego
Lopez-Higuera, Jose Miguel
López-Lorente, Ángela Inmaculada
López-Paz, José Luis
Lorenz, Pascal
Lorenzo, E.
Lou, Hubing
Lou, Xia
Lou, Xinhui
Louarn, Guy
Loukas, George
Loutas, Theodoros
Loutfi, Amy
Loutridis, S.J.
Lovisolo, Lisandro
Lowry, Mark
Lozano, Gabriel
Lu, Frank
Lu, Geyu
Lu, Huaguang
Lu, Jian
Lu, Jiwen
Lu, Min
Lu, Ping
Lu, Shiang-Cheng
Lu, Wei
Lu, Zhong
uczak, Sergiusz
Ludwig, Nicholas G.
Luerssen, Martin
Lughofer, Edwin
Luhmann, Thomas
Luigia, Sabbatini
ukomski, Michał
Lund, Björn
Lundberg, Jan
Luo, Ching-Hsing
Luo, Guang-Sheng
Luo, Jianwen
Luo, Jun
LUO, Xiaowei
Lupi, Cyril
Luque-Heredia, Ignacio
Luštrek, Mitja
Luyckx, Geert
Lv, Ke
Lv, Yi
Lyons, Damian M.
Lyons, Keith
Ma, Anzhou
Ma, Bin
Ma, Bing
Ma, Cheng
Ma, Edmond Dik-Lung
Ma, Guo-Ming
Ma, Houyi
Ma, Huilian
Ma, Jianguo
Ma, Ping
Ma, Qiulin
Ma, Tian-Yi
Ma, Xiang
Ma, Yuan-Ron
Macfarlane, Craig
MacGregor, M.H.
Mach, Pavel
Macias, Elsa
MacKenzie, Roger
MacMillan, Bryce
Macnab, Andrew
Maeda, Isamu
Maehle, Erik
Maetzler, Walter
Maggiali, Marco
Magliulo, Maria
Maglogiannis, Ilias
Magno, Michele
Maier, Norbert M.
Majidi, Carmel
Majumdar, Angshul
Makarov, Denys
Maksoud, Talal
Malara, Pietro
Malinski, Tadeusz
Mallinis, Georgios
Malmendal, Anders
Malocha, Don
Manaf, Asrulnizam Bin Abd
Manamanni, Noureddine
Mancini, Adriano
Mancini, Martina
Mander, John
Mandlburger, G.
Manea, Florica
Manera, Maria Grazia
Manes, Gianfranco
Maneuski, Dima
Mangelinckx, Sven
Mankin, R. W.
Mankodiya, Kunal
Mannens, Erik
Mannini, Andrea
Mansouri, Alamin
Mantatzis, Michalis
Mäntele, Werner
Mao, Hongju
Mao, Qingzhou
Mao, Xuchu
Marco, Ario De
Marcolin, Federica
Mare Srbinovska, Mare
Mariani, Stefano
Marinesco, Stéphane
Marini, Elisabetta
Markowitch, Olivier
Marrazza, Giovanna
Marsh, Eric
Martalo, M.
Martalò, Marco
Martí, Pau
Martin, Angel
Martin, Donald
Martín, Ferran
Martin, Suzanne
Martinelli, Eugenio
Martinez de Dios, José Ramiro
Martinez Dominguez, Francisco
Martínez, Alberto Caballero
Martinez, Jorge L
Martínez, José F.
Martínez-Ballesté, Antoni
Martinez-Perdiguero, Josu
Martinez-Tabares, F. J.
Martin-Gorriz, Bernardo
Martí-Vargas, J.R.
Marusenkov, Andriy
Masamune, Ken
Massa, A.
Massai, Rossano
Matko, Vojko
Matos, Aníbal
Matson, Eric T
Matsushima, Dai
May, Elebeoba E.
Mayaram, Karti
Mazid, Abdul
Mazurek, Przemysław
Mazza, Claudia
Mazzetto, Fabrizio
Mazzoni, Augusto
McEvoy, James
Mcewen, Mark P.
McGibney, Alan
McGrath, Denise
McKeever, Susan
McLeod, Euan
Mcmeekin, Scott
McNeil, Jeremy N.
McPeak, Kevin
Mcshane, Michael
McWilliam, Stewart
Md. Shakaff, Ali Yeon
Me, Gianluigi
Medeiros Ferreira, João Paulo
Meder, Roger
Medved, Vladimir
Meek, Sanford G.
Mei, Alessandro
Mei, Xuesong
Meinecke, Marc-Michael
Meinel, Christoph
Mejias, Luis
Melo, POS Vaz de
Melodia, Tommaso
Memon, Nasir
Mendes, Paulo Mateus
Mendez Zorrilla, Amaia
Menezes, Paulo
Meng, Huadong
Meng, L. F.
Meng, Xu
Meng, Yu Song
Menna, Fabio
Menotti, David
Meola, Carosena
Mera, David
Meratnia, Nirvana
Merla, Arcangelo
Merletti, Roberto
Mesas-Carrascosa, Francisco Javier
Mescheder, Ulrich
Mestrom, Rob
Metsis, Vangelis
Mettler, Bérénice
Meulenberg, Eline P.
Meunier, Jean
Meyer, Carly
Meyer, Thomas
Miao, Jianmin
Miao, Qiang
Miao, Zhenwei
Michail, Konstantinos
Michal, Carl
Michelena, Marina
Micheloni, Mauro
Michelson, Dave
Midtiby, Henrik Skov
Mignani, AnnaGrazia
Mignanti, Silvano
Miguel, Leandro Fleck Fadel
Mihaylova, Lyudmila
Mikada, Hitoshi
Milanova, Fani
Milazzo, Alberto
Milczarek, Grzegorz
Miled, Amine
Milella, Annalisa
Miller, James
Milligan, Graeme
Milosevic, Bojan
Min, Jianyuan
Min, Junhong
Min, Mart
Minakuchi, Shu
Minardo, Aldo
Minematsu, Nobuaki
Mineno, Hiroshi
Minet, Pascale
Mingesz, Robert
Minoni, Umberto
Minor, Mark A.
Miribel, Pedro Luis
Miroshnichenk, Andrey E.
Mirsky, Vladimir M.
Misiakos, Konstantinos
Misron, Norhisam
Missinne, Jeroen
Mitchell, John
Mitchell, Kenneth
Mittlboeck, Manfred
Mitton, Nathalie
Mizsei, János
Mizuhashi, Keiichi
Mizukami, Shin
Mizukami, Yuzo
Mlsna, Todd
Mo, Changki
Mo, Jinhan
Mochi, Iacopo
Modotto, Daniele
Moeslund, Thomas
Moh, Sangman
Mohamed A., Mahgoub
Mohamedi, Mohamed
Mohri, Kaneo
Moisen, Gretchen
Molavi, Behnam
Molina, J.M.
Momm, Henrique G.
Monaghan, David
Monakhova, Yulia
Monekosso, Dorothy
Montavont, Julien
Monteil, Thierry
Montereali, Maria Rita
Montoliu, Raul
Monty, Chelsea
Moore, Richard J. D.
Moradi, Marjan
Morais, Hugo
Moraleda, Alberto Tapetado
Morales, Diego P.
Morandi, Luca
Moreno, M.A.
Morgado, Tiago André Nogueira
Morimitsu, Hidetaka
Morozov, Maxim
Morpurgo, Margherita
Mortari, Alessia
Morze, Cornelius von
Moscato, Vincenzo
Moshou, Dimitrios
Moshou, Dimitris
Mossi, Karla
Motta, Nunzio
Mouradian, Alexandre
Moya, Francisco
Moya, Jose Manuel
Možek, Matej
Muench, Falk
Mugge, Winfred
Muggleton, Jen
Mukherjee, Partha Sarathi
Muldoon, Conor
Muller, Cobus
Müller, Georg
Mulvenna, Maurice
Munishwar, Vikram P.
Munjal, Aarti
Muñoz, Carlos
Muñoz, Roberto
Muñoz-Gea, Juan Pedro
Murakawa, Hideki
Muraleedharan, Rajani
Muroyama, Masanori
Murray, Kevin
Murray, Victor
Muševič, I.
Mussetta, Marco
Mustafa, Besim
Muthuraman, Muthuraman
Myers, Mike
Myers, Oliver J.
Myung, Hyun
Naaman, R.
Nabi, Majid
Nabok, Alexei
Naeem, Usman
Nafarieh, Alireza
Nagamune, Kouki
Nagaraj, Vinay J.
Nagarajah, Romesh
Nagatani, Naoki
Nagy, Zoltán
Naik, Ganesh
Naik, Ganesh R.
Najarian, Kayvan
Nakamiya, Toshiyuki
Nakamura, Hideaki
NAKAMURA, Yoshinobu
Nakane, Akio
Nakano, Kimihiko
Nakata, Makiko
Nakayama, Masaharu
Nalda-Molina, Ricardo
Namiesnik, Jacek
Nanayakkara, Thrishantha
Nanni, Loris
Napieralska Juszczak, Ewa
Naraghi-Pour, Mort
Naranjo, Jose
Natraj, Vijay
Navratil, Martin
Nawaz, Sarfraz
Nedelec, Jean-Marie
Negro, Francesco
Nehorai, Arye
Neil, Daniel
Neild, Adrian
Nele, Márcio
Nemova, Galina
Neri, Ferrante
Neri, Giovanni
Neska, Anne
Neto, P.
Netto, João Cesar
Neves, Paulo A. C. S.
Newman, Kimberly E.
Nex, Francesco
Ng, Kc
Ng, Wee Siong
Nguyen, Hoang
Nhan, Nguyen-Thanh
Ni, Bingbing
Niazi, Imran Khan
NICOLA, Mario
Niederleithinger, Ernst
Nielsen, Shawn
Nieminen, Heikki
Nievinski, Felipe
Nijholt, Anton
Nijland, Wiebe
Nikita, Konstantina
Nikolic, Janosch
Niku, Saeed
Ning, Huansheng
Nirjon, Shahriar
Nishiuchi, Nobuyuki
Nitti, Michele
Niu, Shengjie
Niu, Xiaoji
Nocita, Marco
Nojima, Yusuke
Nomura, Takanori
Norman, Kent
Norris, Michelle
Norton, Michael Louis
Novais, Paulo
Novak, Domen
Novakovic, Jelica
Novella, José Ignacio Moreno
Novoa, Stefani
Nowotny, Thomas
Nüchter, Andreas
Nugen, Sam R.
Nunes Nogueira, Rogério
Nunes Rodrigues, Genaína
Nunes, Maria Fernanda de Oliveira
Nyka, Krzysztof
Nymoen, Kristian
Nyokong, Tebello
O’Mahony, Conor
O’mahony, Fergal
Oberti, Roberto
Ochoa, Sergio
Ocvirk, Gregor
Oddo, Calogero
Odgaard, Peter Fogh
O'Driscoll, Cillian
Oelkrug, Dieter
Oestges, Claude
O’Grady, Michael
Oh, Min-Cheol
Oh, Tong In
Ohodnicki, Paul R.
Ohtani, Toshihiro
Oikonomou, George
Okawai, Hiroaki
Okparanma, Reuben
Olaizola, Santiago Miguel
Olaru, Andrei
Olchowik, Jan M.
Oleinick, Alexander
Oligeri, Gabriele
Olivares, Alberto
Olivares, Teresa
Oliveira, Luís
Oliveira, Sérgio Campello
Oliver, Gabriel
Oliver, Nick
Oliver, Probst
Olivier, Pierre
Olsen, Michael J.
Olsen, Rasmus
Olsson, Roger
Olthuis, W.
Olyaee, Saeed
Omrani, Hichem
Onen, Onursal
Onoe, Morio
Oprea, Alexandru
Ordóñez, Fco. Javier
Orglmeister, Reinhold
Orozco, Jahir
Orte, Angel
Ortiz, Alberto
Ortolani, Marco
Ortuno, Joaquín A.
Osaki, Toshihisa
Osborn, Jon
Osborne, R.
Osowski, Stanisław
Ostasevicius, Vytautas
Ostendorf, Bertram
Otoom, Ahmed Fawzi Ali
Ou, Jianzhen
Ou, Shumao
Oude Elberink, S.J.
Oujja, Mohammed
Own, Chung-Ming
Ozog, Paul
Padmanabhan, Santhosh
Page, Wyatt
Pagiatakis, Spiros
Paja, Wieslaw
Pal, Sudeshna
Palacín, Jordi
Palego, Cristiano
PALMIERI, Luca
Palomares, Jose Manuel
Palumbo, Filippo
Palzer, Stefan
Pan, Anlian
Pan, Chen
Pan, Leiqing
Pan, Liyang
Pan, Meng-Shiuan
Pan, Tung-Ming
Pan, Xianfei
Pan, Zengxi
Pang, Changhyun
Panneton, Bernard
Pantazis, Dimos N.
Pantelopoulos, Alexandros
Paolini, Enrico
Papa, Joao Paulo
Papadourakis, George
Papaelias, Mayorkinos
Papaioannou, Athanasios
Papapetrou, Panagiotis
Papaspiliopoulos, O.
Paradiso, Rita
Parajuli, Suman
Pardalos, Panos
Park, Chan Gook
Park, Gyuhae
Park, H. S.
Park, Jae-Hyoung
Park, Jihye
Park, Joon-Sik
Park, Jungmin
Park, Kwang Suk
Park, Kyung-Soo
Park, Mi Kyoung
Park, Myong-Soon
Park, Sehyun
Park, Tae Jung
Park, Tai Hyun
Park, YongKeun
Park, Yong-Lae
Parkkola, Riitta
Parsons, Gregory N.
Passian, Ali
Passieux, Jean-Charles
Pastorino, Matteo
Pathan, Al-Sakib Khan
Patias, Petros
Patimisco, Pietro
Patrono, Luigi
Patron-Perez, Alonso
Patskovsky, Sergiy
Pattichis, Marios S.
Paul, Diby
Paulson, Kevin
Pauwels, Karl
Pavliček, Pavel
Pavlidis, George
Pavlidis, Ioannis
Paya, Luis
Paydar, O.H.
Pearce, Joshua M.
Pearton, Stephen J.
Pecorella, Tommaso
Peeters, Marloes
Pei, Ling
Pei, Songwen
Pejcic, Bobby
Pelayo, Francisco
Peng, Chien-Fang
Peng, Hanjng
Peng, Lihui
Peng, Yucheng
Penha, Rui
Penmatsa, Varun
Pennacchi, Paolo
Pennazza, Giorgio
Perales, Fco J.
Perallos, Asier
Pereira, Rubem
Perera, Lokukaluge P.
Peres, António Manuel
Perez, Alfredo J.
Pérez, Gonzalo Wangüemert
Perez, Manuel
Perez, Rodrigo
Perez, Rosa
Pérez-Cabré, Elisabet
Perez-Ruiz, Manuel
Perez-Uribe, Andres
Pertilä, Pasi
Pertusa Ibáñez, Antonio
Pertuz, Said
Pescaru, Dan
Peteinatos, G. G.
Peter, Hubinský
Peter, Steffen
Petersen, Stig
Petit, Gérard
Petit, Nicolas
Petovello, Mark
Petroccia, Roberto
Pfiffner, Flurin
Pham, Congduc
Phan, Manh-Huong
Philip, Nada
Philippides, Andy
Phillips, Alex
Piano, Martina
Picking, Richard
Picone, Marco
Picou, Erin Margaret
Piérard, Sébastien
Pierce, David
Pierrot-Deseilligny, Marc
Piester, Dirk
Pignaton de Freitas, Edison
Pijanowska, Dorota G.
Pilloni, Virginia
Pilny, L.
Pimenta, Tales Cleber
PINGARRON, Jose Maria
Pinto, Andry Maykol
Pinto, Paula C.A.G.
Piras, Andrea
Pires, J. Norberto
PIRO, Benoit
Pirotti, Francesco
Pirozzi, Salvatore
Pirro, S
Pissadakis, Stavros
Pistohl, Tobias
Plant, Richard E.
Plant, Thomas K.
Plasqui, Guy
Pławiak, Paweł
Pleitez, Miguel
Poenar, Daniel P.
Poggi, Agostino
Pohanka, Miroslav
Poletkin, Kirill
Politis, Chistos
Pollisiou, Moschos
Polo, José
Pomalaza-Ráez, Carlos
Popovics, John S.
Poppe, László
Porchetta, Alessandro
Portilla, Jorge Higuera
Portugal, Paulo
Poslad, Stefan
Postolache, Octavian
Potortì, Francesco
Pötsch, Thomas
Potter, Mark
Pottie, Greg
Pottier, Bernard
Pourhomayoun, Mohammad
Pourkamali, Siavash
Pourtakdoust, S H
Powers, David
Powers, Robert
Prades, Joan Daniel
Prakash, Ranganathan
Prats, Mario
Preuveneers, Davy
Prevost, Alexis
Prida, V. M.
Prodromidis, Mamantos
Proença, Hugo
Scampicchio, Matteo
Prytherch, David
Psiaki, Mark L.
Pu, Fang
Pugi, Luca
Puig, Luis
Puig, Vicenc
Puliafito, A.
Pursula, Pekka
Puttonen, Eetu
Pyeon, Cheolho
Pyk, Pawel
Pyka, Krystian
Qian, Kemao
Qian, Kun
Qin, Faxiang
Qin, Huabiao
Qin, Jianwei
Qiu, Jian-Ding
Qiu, Jinhao
Qiu, Liang
Qiu, Ying
Qiu, Zhi-Cheng
Qu, Peng
Quaglia, Davide
Queneau, Yves
Raahemifar, Kaamran
Rachkidy, Nancy El
Radecki, Jerzy
Radosavljevic, Goran
Radu, Fechete
Radwan, Essam
Raghavendra, Ramachandra
Ragni, D.
Rahamn, Masudur
Raimundo Jr, Ivo
Ram, Manoj K.
Ramanavicius, Arunas
Rambla-Alegre, Maria
Rana, Dipak
Ranasinghe, Damith
Rao, Ashwin
Rao, Singamaneni S.
Raposo, M. Manuela M.
Rapp, Michael
Rappoport, Josh
Raptis, Ioannis
Rashkovska, Aleksandra
Rasooly, Avraham
Rasson, Jean L
Rau, Ruey-Juin
Rauber, Thomas W.
Ravanbakhsh, Mehdi
Ray, Jim
Raya, José G.
Razansky, Daniel
Razzaque, Mohammad Abdur
Realini, Eugenio
Redding, Brandon
Redmond, Steven
Redondi, Alessandro
Redondo, R.
Reen, Jerry
Reenalda, Jasper
Regan, Amelia C.
Regazzoni, Carlo S.
Reger, Johann
Rehman, Ihtesham
Reich, Siegfried
Reig, Càndid
Reinoso, Oscar
Reis, Manuel J.C.S.
Reisslein, Martin
Rekand, T.
Remoundaki, E.
Ren, Dahai
Ren, Liyong
Renaudin, Valérie
Renero-Carrillo, Francisco-J.
Repo, Tapani
Reshetilov, Anatoly N.
Residori, Stefania
Resop, Jonathan
Rettberg, Achim
Reverchon, Sylvie
Revuelta, Francisco Florez
Reza, Ahmed Wasif
Rhee, Huinam
Rhyu, Se-Hyun
Riahi, Reze
Ribeiro, Ángela
Ribés, A.
Ricchiuti, A. L.
Ricci, Giuseppe
Ricciardi, Serena
Riccobene, Giorgio
Richelli, Anna
Richter, Lars
Ricketts, David S.
Riedmaier, Irmgard
Riffell, Jeff
Rijal, Hom
Rimeika, Romualdas
Rinaldi, Stefano
Rinaudo, Fulvio
Ripka, Pavel
Ripper, Gustavo
Rist, Thomas
Ritsch-Marte, Monika
Riziotis, Christos
Rizvi, Syed
Rizzolo, A.
Roalter, Luis
Roberts, Gethin
Roberts, Mark E.
Robertson, Duncan Alexander
Robertson, Will
Robertson, William M.
Rocchi, A.
Rocha, Luis
Rocha, Luís Alexandre
Rodger, James A.
Rodolà, Emanuele
Rodrigues, Carlos
Rodríguez, Francisco
Rodriguez, Joel
Rodriguez, Pedro A.
Rodríguez-Esquerre, V. F.
Rodriguez-Gonzalvez, Pablo
Rodríguez-Molina, Jesús
Rogers, John A.
Rogier, Hendrik
Roh, Kyoung-Min
Rohac, Jan
Roig, Ignacio Bosch
Rojas, Alexander Malaver
Rojas, Luis Martin
Roman, Adam
Roman, Chris
Romani, Aldo
Romanovas, Michailas
Rommers, G.M. (Clemens)
Rong, Qiangzhou
Ronkainen, Niina J.
Rönnholm, Petri
Rosa, Joaquín
Rose, Joseph L.
Rosenberger, M.
Rosina, Elisabetta
Rosolem, J
Rossi, Stefano
Rothkrantz, Leon
Rotondo, Damiano
Roufarshbaf, Hossein
Roundy, Shad
Routen, Ash
Rovira-Más, Francisco
Rozlosnik, Noemi
Rubini, Riccardo
Rubio, Jose Manuel Amigo
Rubio-Bollinger, Gabino
Ruckelshausen, Arno
Rudenko, M.I.
Rudnitskaya, Alisa
Rudolph, Joachim
Rudzicz, Frank
Ruegamer, Alexander
Ruizenaar, Marcel
Ruiz-Fernández, Luis Ángel
Ruiz-Sandoval, Manuel
Ruo, Yuan
Ruotsalainen, Laura
Ruzgas, Tautgirdas
Rygielski, Piotr
Saad, Walid
Saal, Hannes
Sabatini, Angelo
Sabol, Donald
Sachau, Delf
Sadegh Amiri, Iraj
Sadhu, Ayan
Sadr, Alireza
Saey, Timothy
Safacas, Athanasios
Saffar, Saber
Sahoo, Prasan Kumar
Sainz, Beatriz
Sakaki, Taisuke
Sakaki, Toshihiro
Salacinski, Tedeusz
Salas, Nelson
Salazar, Félix
Salazar-Torres, Jose J.
Salem, Osman
Salinas, Rosario
Salm, Cora
Salmain, M
Salsbury, Fred
Samek, Wojciech
Samitier, Josep
Sampalli, Srinivas
Sampietro, Daniele
Sams, Thomas
Samson, Guy
Samuel, Raymond
Sánchez Montero, David
Sánchez, David
Sanchez, Manuel Garcia
Sanchez, Nilda
Sanders, David
Sang, Liwen
Sankaran, Sindhuja
Sankarraj, Anand
Sanli, Abdulkadir
Sanmartín, Patricia
Santana, Modesto Castrillón
Santarelli, Giorgio
Sante, R. Di
Santhanagopalan, Sunand
Santiago-Aviles, Jorge J.
Santos, Luís Paulo Peixoto Dos
Santos, Vitor
Santulli, Carlo
Sanyal, Jibonananda
Sanz, J.
Saponara, Sergio
Sardini, Emilio
Sarkar, Santanu
Sasamoto, Akira
Sato, Kazuo
Sato, Yukari
Satriano, Cristina
Saudreau, Marc
Sauerbier, Martin
Savage, Nick
Savage, Paul G.
Sawacha, Zimi
Sawan, Mohamad
Sawyer, Shayla
Saxén, Henrik
Saxena, Navrati
Sayago, Isabel
Sberveglieri, Veronica
Scaioni, Marco
Scalenghe, Riccardo
Scarpa, Fabrizio
Schade, Wolfgang
Schaferling, Michael
Scheller, Frieder W.
Schepers, James S.
Schindelhauer, Christian
Schirhagl, Romana
Schizas, Ioannis
Schmid, Natalia
Schmidt, Uwe
Schön, Steffen
Schultz, Stephen
Schultz, Zac
Schumacher, Jens
Schumacher, Thomas
Schurig, Thomas
Schuster, Ute
Scilingo, Enzo Pasquale
Scioscia, Floriano
Scognamiglio, Viviana
Seatovic, Dejan
Sebastian, Jose Maria
Seco-Granados, Gonzalo
Segawa, Hiroyo
Segl, Karl
Seidel, Dominik
Seidel, Sally
Seinturier, Lionel
Sejdić, Ervin
Selekwa, Majura F.
Sellers, Eric W
Semenova, Yuliya
Sencadas, Vitor
Sendra, Sandra
Senesi, Giorgio S.
Sengupta, Anupam
Seo, Jin Keun
Sepúlveda, Borja
Sequeira Gonçalves, Paulo Jorge
Sequeira, João
Serio, Carmine
Serizel, Romain
Seron, Maria
Serral, Estefanía
Serrano, Emilio
Serranti, Silvia
Sethi, Ricky J.
Seung-Bok, Choi
Sexidis, Lazaros A.
Seyedi, MH
Shabayek, Rahman
Shafait, Faisal
Shafiei, Mahnaz
Shah, Nilam
Shahid, Adnan
Shan, Wanliang
Shangguang, Dihua
Shao, Hong-Bo
Shao, Ling
Shao, Liyang
Shao, Xi
Sharifi, Farrokh J.
Sharma, Mak
Shell, Jethro
Shemshad, Javad
Shen, Huiliang
Shen, Ju
Shen, Jun-Hong
Shen, Sheng-Chih
Shen, Yanfeng
Shen, Yuan
Shen, Yuzhong
Shi, Guangyi
Shi, Jing
Shi, Peng
Shi, Wei
Shi, Weisong
Shibusawa, S.
Shieh, Wann-Yun
Shih, Po-Jen
Shih, Wen-Pin
Shikida, Mitsuhiro
Shim, Byonghyo
Shimabukuro, Yosio
Shimizu, Ken D.
Shin, Hyunchol
Shin, Hyung Shik
Shin, Jitae
Shin, Kyu-Ho
Shin, Yong-beom
Shine, J.M.
Shing Ching, Tak
Shinohara, Yasuo
Shon, Taeshik
Shorter, Joanne H.
Shoshany, maxim
Shu, Lei
Shukla, Nagesh
Shull, Pete B.
Sibirny, A. A.
Sicari, Sabrina
Sidén, Johan
Sidorov, Andrei
Siebert, Frank-André
Sigrist, Markus Werner
Sikka, Pavan
Sillanpaa, Mika
Silva, Ricardo
Silverman, Harvey F.
Silvoni, Stefano
Sim, Khe Chai
Simic, Milan
Simon, Camille
Simonov, Mikhail
Sin, Mandy
Singh, Rituraj
Singhal, Mukesh
Sintim, Herman O.
Sixdenier, Fabien
Skladal, Petr
Skomorowski, Marek
Skrzypczyński, Piotr
Slegers, P.M.
Sleigh, Jamie
Slobodian, Petr
Slonecker, Terrence
Smeaton, Alan
Smietana, Mateusz
Smirnoff, Nicholas
Smith, Bryan
Smith, David
Snoussi, Hichem
Snow, Nicholas
So, Hing-Cheung
Soares, Vasco N.G.J.
Soh, Cheong Boon
Sohail, Manzar
Sohgawa, Masayuki
Soiza, Roy
Soldano, Caterina
Soleimani, Manuchehr
Soleimanpour, Amir Masoud
Soleymani, Leyla
Sołoducho, Jadwiga
Soloviev, Mikhail
Soltanieh, Mohsen Kalantari
Somanathan, Ratnasamy
Somani, Arun
Son, Hungsun
Song, Aiguo
Song, Gangbing
Song, Lijun
Song, Minkyu
Song, Quanjun
Song, Soonho
Song, Xuan
Song, Yang
Song, Yanlin
Song, Zhan
Soon, Benjamin
Soriano, Julio Gómez
Sotiriadis, Stelios
Sotiriou, Georgios
Spagnolo, Giuseppe Schirripa
Spagnolo, Vincenzo
Spaich, Erika
Spanoudakis, Nikolaos I.
Spencer, Billie F
Spinsante, Susanna
Spivak, Arthur
Spletzer, John
Spognardi, Angelo
Spreer, Wolfram
Sprinzl, Mathias
Spüler, Martin
Spurr-Michaud, Sandra J.
Stachowiak, Dorota
Stafylopatis, Andreas-georgios
Stanacevic, Milutin
Stanco, Filippo
Stanislaw, Drobniak
Stanton, Robert
Stauff, Devin L.
Stavropoulos, Thanos G.
Stavroulakis, Georgios E.
Stechele, Walter
Stefani, Alessio
Steffen, Holger
Steil, Garry M.
Steiner, Gerald
Steiner, Matthew
Stergiou, Dimitris
Stevens, Ann M.
Stiebig, Helmut
Stiros, Stathis
Stobiecka, Magdalena
Stoffregen, Thomas
Stoica, Peter
Stojanović, Goran
Stojmenovic, Ivan
Stonehouse, Nicola
Stoyanov, Todor
Stoytcheva, Margarita
Straka, Ondřej
Strano, Michael S.
Strasser, Reto J.
Strehlitz, Beate
Strelcov, Evgheni
Struk, Przemyslaw
Strumiłło, Paweł
Strzelecki, Michat
tumberger, Bojan
tumberger, Gorazd
Su, Fei
Su, Kuo-Lan
Su, Ming-Shou
Su, Pi-Guey
Su, Steven
Suarez, Alvaro
Sudhölter, Ernst J.R.
Sugano, Masashi
Sugano, Shigeki
Sugita, Naohiko
Suh, Doug Young
Suh, Junghae
Suh, Sang-Jin
Suh, Young Soo
Sujit, P.
Sullivan, Pierre E.
Sumer, Bilsay
Sumetsky, Misha
Sun, Chi-Kuang
Sun, Guanghui
Sun, Hongjian
Sun, Hongzhi
Sun, Jinwei
Sun, Lei
Sun, Nai-Hsiang
Sun, Qingquan
Sun, Qizhen
Sun, Shaohui
Sun, Shuli
Sun, Stella
Sun, Weifeng
Sun, Weiqing
Sun, Xiudong
Sun, Yi
Sun, Zhi
Sundramoorthy, Ashok
Susi, Melania
Suwarno, Stanislaus Raditya
Suzuki, Hiroaki
Suzuki, Hiroyuki
Suzuki, Hodaka
Suzuki, Taro
Sveda, Miroslav
Svir, Irina
Swami, Nathan
Swami, Nathan S.
Swartz, R. Andrew
Sweeney, Kevin
Synnes, Kåre
Sysoev, Victor V.
Szeghalmi, Adriana
Székely, György
Szewczyk, R.
Szpytko, Janusz
Szulczewski, Gregory
Szypłowska, Agnieszka
Tadigadapa, Srinivas
Taheri, F.
Takahashi, Chie
Takahashi, Eiji
Takahashi, Ryo
Takamura, Yuzuru
Takeda, Ryo
Takewaka, Satoshi
Takuma, Takashi
Talantzis, Fotios
Tam, Hwayaw
Tamás, Levente
Tamura, Hiroki
Tamura, Toshiyo
Tan, Kun
Tan, Liansheng
Tan, Su-Yin
Tan, U-Xuan
Tan, Yen Kheng
Tanaka, Hiroshi
Tanaka, Tsuyoshi
Tang, Dianping
Tang, Fei
Tang, Liqiong
Tang, Meng-Xing
Tang, Shijun
Tang, Wenlong
Tang, Xinhua
Taniguchi, Akiyoshi
Taniguchi, Masateru
Tao, Dacheng
Tao, Fei
Tao, Junliang
Tao, Lei
Tao, Lili
Tao, Shiquan
Tao, Xiaoming
Tapia, Dante
Tapia, Emmanuel
Tardif, Pierre Martin
Tasis, Dimitrios
Tateishi, Ryutaro
Taubin, Gabriel
Tavío, María del Mar
Tayade, Rajesh J.
Tchrakian, Tigran
Teixeira, Bruno O.S.
Teixeira, Marcos F.S.
Telem, Gili
Teles, Fernando Sérgio Rodrigues Ribeiro
Telfer, Scott
Telkki, Ville-Veikko
Téllez Zenteno, José F.
Tello, Marivi
Tel-Vered, Ran
Temko, Andriy
Tenedorio, Jose Antonio
Teng, Rui
Tepe, Kemal Tepe
Testoni, Nicola
Teunissen, P.J.G.
Teza, Giordano
Thakor, Nitish
Thessler, Sirpa
Theuwissen, Albert J. P.
Thewlis, Dominic
Thiel, David
Thielemann, Christiane
Thiran, Patrick
Thomae, Enrico
Thomson, Steven J.
Thoonen, G.
Thouand, Gerald
Thoury, Mathieu
Tian, Fengchun
Tian, Guiyun
Tian, Wei-Cheng
Tian, Yu
Tidoni, Emmanuele
Tie, Li
Tiloca, Marco
Timmermann, Dirk
Timotheou, Stelios
Timpe, Shannon J
Ting, Lena H.
Tisan, Alin
Tisch, Ulrike
Tisseyre, Bruno
Tittel, Franck
Tjin, Swee Chuan
Tofighi, Mohammad
Tognetti, Alessandro
Tollefsen, Dag
Tomarchio, Orazio
Tomás, Juan
Tomasi, Riccardo
Tomažič, Sašo
Tomkiewicz, Dariusz
Tommaselli, Antonio M. G.
Tong, XingLin
Tong, Yan
Toniolo, Rosanna
Tonokura, Kenichi
Torrens, Francisco
Torres, Arnau Mir
Torres, David
Torres, Miguel
Torri, Luisa
Torsi, Luisa
Tosi, Daniele
Touhafi, Abdellah
Tran, Binh Q.
Tran, Gia Khanh
Traver, Vicente
Travieso González, Carlos Manuel
Treiber, Martin
Trevathan, Jarrod
Triantafilis, John
Tricoli, Antonio
Trinca, D
Trogl, Josef
Trommer, Gert F.
Tron, Roberto
Troncoso, Carmela
Troni, Giancarlo
Trono, Cosimo
Trost, Stewart G.
Trusov, A. A.
Trzynadlowski, Andrzej M.
Tsagkatakis, Grigorios
Tsai, Du-Ming
Tsai, Meng-Tsan
Tsai, Miching
Tsai, Tsung-Nan
Tsai, Wen-Chi
Tsakalof, A
Tsalatsanis, Athanasios
Tsarev, Andrei
Tsay, Chien-Yie
Tse, Norman
Tse, Peter
Tsekouras, George E.
Tseng, W. L.
Tsiligiridis, Theodore A.
Tsotsis, Theodore T.
Tsourveloudis, N.
Tsui, Po-Hsiang
Tu, Rui
Tudor, John
Tur, Moshe
Tura, Andrea
Turcza, Pawel
Turner, Ken
Tuzlukov, Vyacheslav
Tzallas, Alexandros
Uchida, Takahiro
Uchikoba, Fumio
Uddin, Shahadat
Ueno, Yuko
Ukkonen, Leena
Ulacha, Grzegorz
Ullah, Sana
Umasankar, Yogeswaran
Unger, Michael A.
Uno, Shigeyasu
Urban, Bodo
Urbann, Oliver
Urzaiz, Jose Gabriel
Usamentiaga, Rubén
Uslander, Thomas
Ußmüller, Thomas
Usuda, Takashi
Vaezi, Michael F.
Vainio, Markku
Valcárcel, Miguel
Valcke, Roland
Valera, Enrique
Valls, Xavier Binefa
van den Broek, Egon L.
van den Noorta, Josien C.
van der Werff, Harald
van Eerdenburg, Frank J C M
van Hees, Vincent
van Putten, Michel J.A.M.
van Rienenb, Ursula
Van Someren, Maarten
Van, Vien
Vandenbossche, Jochen
Vanemon, Jeanette
Vanli, O.Arda
Varaiya, Pravin
Vardasca, Ricardo
Vardy, Andrew
Vargas, César
Varnum, Susan
Vasilakos, Athanasios V.
Vasileiadis, M.
Vastarella, Walter
Vázquez Bermúdez, David
Vega Pérez, Gracia
Vehkaoja, Antti
Vela, Patricio Antonio
Velotta, Raffaele
Veltink, Peter H.
Veluvolu, Kalyana C.
Venchi, Giuseppe
Venkatasubramanian, Krishna
Venticinque, Salvatore
Vergallo, Patrizia
Vergara, Ekhiotz
Verhoeven, Geert
Verikoukis, Christos
Verma, Anoop
Vesel, Alenka
Vetter, Stefan W.
Vettore, Antonio
Vichi, Francesca
Vidal-Verdu, Fernando
Vidic, Jasmina
Viegas, Jaime
Vieira, Marco
Vieri, Marco
Viharos, Zsolt János
Villarreal, Vladimir
Villez, Kris
Villmann, Thomas
Vincenzi, Loris
Vinel, Alexey V.
Viollet, Stéphane
Vipul, Lugade
Visconti, Fernando
Vitabile, Salvatore
Vítek, Martin
Vlacic, Ljubo
Voiculescu, Ioana
Volosyak, Ivan
Volz, Jürgen
Vona, Marsette
Vonikakis, Vassilios
Voss, Andreas
Vosselman, George
Vougioukas, Stavros G.
Vouros, Paul
Vrazic, Mario
Vultaggio, Mario
Vural, Serdar
Wac, Katarzyna
Wade, Scott
Wagner, Bernardo
Wahid, Khan A.
Wakin, Michael B.
Walczykowski, Piotr
Waldvogel, Siegfried R.
Walgraeve, Christophe
Walker, Jacqueline
Walsh, Kerry
Walter, Jonathan P
Wan, Kai Tai
Wan, Yuan
Wang, Abraham
Wang, Aimin
Wang, Bang
Wang, Bin
Wang, Bo
Wang, Ce
Wang, Chao
Wang, Chaoming
Wang, Chaonan
Wang, Chung-Ho
Wang, Chunyu
Wang, Dian Hui
Wang, Dong
Wang, Fei
Wang, Feng
Wang, Gou-Jen
Wang, Guoquan
Wang, Hai
Wang, Hongmei
Wang, Hongwu
Wang, Hua
Wang, Huaqing
Wang, Huaxiang
Wang, Jianguo
Wang, Jian-Neng
Wang, Jianqi
Wang, Jianqiang
Wang, Jing
Wang, Jingchuan
Wang, Jinling
Wang, J-J
Wang, Juanle
Wang, Jun
Wang, Junbo
Wang, Keping
Wang, Keyi
Wang, Lei
Wang, Lili
Wang, Ling
Wang, Linwei
Wang, Pengfei
Wang, Pi-Chung
Wang, Ping
Wang, Qi
Wang, Qian
Wang, Qiang
Wang, Qin
Wang, Qing
Wang, Ruixuan
Wang, Shaopeng
Wang, Sheng-Shih
Wang, Shuenn-Jyi
Wang, Shuo
Wang, Tao
Wang, Victor
Wang, Wei
Wang, Wen
Wang, Wen-Qin
Wang, Xianqiao
Wang, Xiaona
Wang, Xihua
Wang, Xin
Wang, Xingwei
Wang, Xinheng
Wang, Xu-Dong
Wang, Xuelin
Wang, Yan
Wang, Yaojin
Wang, Yao-Tien
Wang, Yi
Wang, Ying
Wang, Yixin
Wang, Yong
Wang, Yucai
Wang, Yuelin
Wang, Yuhong
Wang, Yujie
Wang, Yun
Wang, Zeneng
Wang, Zhe
Wang, Zhengang
Wang, Zhenxin
Wang, Zheyao
Wang, Zheyu
Wang, Zhiwei
Ward, Michael
Wark, Alastair W.
Warren, Steve
Warriach, Ehsan Ullah
Wartzek, Tobias
Wasisto, Hutomo Suryo
Watanabe, Yuji
Waters, Chris
Watras, Carl
Watteyne, Thomas
Webster, John
Webster, Robert S.
Wechsler, Harry
Wee, Ser
Wei, Liangming
Wei, Shun-Jun
Wei, Tao
Wei, Yang
Weichert, Franck
Weidemann, Alan
Weimann, Claudius
Weinberg, Graham V
Weinberg, Marc
Weis, Stephen
Weiss, Pierre
Welter, John T.
Wen, Chih-Yu
Wen, Weijia
Wen, Yicheng
Wencel, Dorota
Werneck, Marcelo
Werner, Jürgen H.
Werner, Martin
Westland, Stephen
Weyn, Maarten
Whelan, Matthew J.
White, Ryan
Whitmore, Stephen A.
Wicks, Michael
Wickström, Nicholas
Wiegärtner, Sven
Wielgosz, Pawel
Wilksch, Philip
Willander, Magnus
Willers, Jeffrey
Williams, Billy
Williams, Kathryn R.
Wilson, Benjamin
Winkelbach, Simon
Wismüller, Roland
Wisniewski, Marcin
Witczak, Marcin
Withayachumnankul, Withawat
Witrisal, Klaus
Wittenberg, Nathan
Wittstock, Gunther
Wojkiewicz, J.L.
Wolbrecht, Eric
Wolfbeis, Otto S.
Wolinski, Tomasz R.
Wollenberger, Ulla
Won, Chee-Sun
Won, Jong-Hoon
Wong, Alexander
Wong, Danny K. Y.
Wong, K. Thomas
Wong, Man-kin
Wong, Sebastien
Wong, Tak Sing
Wong, Yiu-Chung
Wongchoosuk, Chatchawal
Wood, Stephen L.
Wright, Matthew
Wu, Bin
Wu, Chao-Cheng
Wu, Dajian
Wu, Fan
Wu, Gang
Wu, Jiaji
Wu, Joseph
Wu, Kangbing
Wu, Lifeng
Wu, Nan
Wu, Nan-Jian
Wu, Qi
Wu, Qiang
Wu, Quincy
Wu, Ren-Jang
Wu, Ruiheng
Wu, Shin-Tson
Wu, TongHai
Wu, Wei-chiang
Wu, Xiaoming
Wu, Xiaopei
Wu, Xinjun
Wu, Xue-zhong
Wu, Yik-Chung
Wu, Yuanming
Wu, Zheng
Wu, Zhifang
Wujcik, Evan K.
Würtz, Rolf P.
Wynter, Laura
Wysocki, Gerard
Wysocki, Laura
Xanthopoulos, Petros
Xi, Jiangtao
Xi, Yong
Xia, Andong
Xia, Bin
Xia, Ke Yu
Xia, Linyuan
Xia, Na
Xia, Yuanqing
Xiang, Yang
Xiang, Zhiyu
Xiao, Hai
Xiao, Yang
Xiaobo, Zou
Xie, Gaogang
Xie, Hongbo
Xie, Hua
Xie, Pu
Xie, Xing
Xie, Zhijian
Xiong, Jijun
Xiong, Jiping
Xiong, Yongliang
Xu, Chuanlong
Xu, ChunXiang
Xu, Fei
Xu, Feng
Xu, Guochang
Xu, Hongli
Xu, Jiangtao
Xu, Jingkun
Xu, Liang
Xu, Mingsen
Xu, Peiliang
Xu, Richard
Xu, Sen
Xu, Wenyao
Xu, Yong
Xu, Yongjun
Xuan, Fu-Zhen
Xue, Bindang
Xue, Ning
Xue, Wei
Yakes, Betsy
Yamada, Usuke
Yamamoto, Kazukiyo
Yamane, Katsu
Yamato, Masayuki
Yamazaki, Tatsuya
Yan, Cheng
Yan, Mingyuan
Yan, Na
Yan, Ruqiang
Yan, Tianhong
Yan, Xijun
Yan, Yongfeng
Yáñez-Sedeño, Paloma
Yang, Bai
Yang, Chaowei
Yang, Chia-Min
Yang, Chungang
Yang, Dingtian
Yang, Fan
Yang, Gelan
Yang, Guang-Hong
Yang, Hee-Deok
Yang, Hong-Chang
Yang, Hsiharng
Yang, Jie
Yang, Jun
Yang, Kun
Yang, Lian X.
Yang, Liang
Yang, Ling
Yang, Ming
Yang, S.H.
Yang, Seong Wook
Yang, Shikuan
Yang, Wuqiang
Yang, Xiaodong
Yang, Xiaoming
Yang, Ya-Ting
Yang, Yi
Yang, Yongfeing
Yang, Yuh-Shyong
Yang, Zhengpeng
Yang, Zhijun
Yao, Leeh-Ter
Yao, Shanshan
Yao, Suying
Yap, Wai Kean
Ye, Baoxian
Ye, Jianshan
Ye, Juan
Ye, Mao
Yeh, Bih-Chyun
Yen, C.W.
Yen, Jia-Yush
Yeo, Hyeop-Goo
Yerong, Yerong
Yevgeni, Koucheryavy
Yi, Chih-Wei
Yi, Jiabao
Yi, Jingang
Yi, Tinghua
Yi, Yonghong
Yiallourou, Stephanie
Yin, Shen
Yin, Wuliang
Ying, Ji Lian
Ying, Liming
Yoe, Hyun
Yokoyama, Yoshihito
Yong, Fang
Yong, Yang-Chun
Yoo, Jung-Woo
Yoo, Seong-Eun
Yoo, Seong-moo
Yoon, Euisik
Yoon, Gilwon
Yoon, Juyoung
Yoon, Seokhoon
Yoshimura, Takeshi
Yoshioka, Takeshi
You, Qinglong
Young, Rupert
Young, Stephen
Young, William
Yu, Chin-Ping
Yu, Dejie
Yu, Haining
Yu, Hongmei
Yu, Hongyu
Yu, Huang
Yu, Jiguo
Yu, Kook-Hyun
Yu, Le
Yu, Ming
Yu, Qian
Yu, Ruiyun
Yu, Shui Yu
Yu, Wei
Yu, Xiang
Yu, Xiong
Yu, Yuebin
Yu, Yunyue
Yuan, Feiniu
Yuan, Guangwei
Yuan, Junsong
Yuan, QiangQiang
Yuan, Ruo
Yuan, Weizheng
Yuan, Ze-Chun
Yuen, Peter
Yufeng, Zhou
Yun, Kwang-Seok
Yupapin, Preecha
Yusa, Noritaka
Yvert, Blaise
Zabulis, X
Zaeh, Michael
Zagar, Bernhard
Zaharis, Zaharias D.
Zakharov, Yuriy
Zampella, Francisco
Zampetti, E.
Zan, Hsiao-Wen
Zanella, Andrea
Zappatore, Marco
Zarco-Tejada, P.J.
Zarpelão, Bruno
Zelenovskiy, P. S.
Zeng, Hong
Zeng, Hulie
Zeng, Rong
Zeng, Tao
Zeng, Xiaoqiao
Zentgraf, Ulrike
Zevenbergen, Marcel A. G.
Zhang, Baoxian
Zhang, Caiyun
Zhang, Changjiang
Zhang, Cheng
Zhang, Chris
Zhang, Chunhua
Zhang, Deyu
Zhang, Dongzhi
Zhang, Guanglie
Zhang, Guangming
Zhang, Guo
Zhang, Guohui
Zhang, Haihong
Zhang, Haijun
Zhang, Hao
Zhang, Houxiang
Zhang, Huihui
Zhang, Jiantao
Zhang, Jin
Zhang, Jinglan
Zhang, Jixi
Zhang, Jixian
Zhang, Joy Ying
Zhang, Jue
Zhang, Julie
Zhang, Jun
Zhang, Lei
Zhang, Liangpei
Zhang, Lifeng
Zhang, Lu
Zhang, Miluo
Zhang, Pei
Zhang, Qijin
Zhang, Qiqing
Zhang, Shengping
Zhang, Shigeng
Zhang, Shujun
Zhang, Tao
Zhang, Tong
Zhang, Weigang
Zhang, Weiping
Zhang, Wen-Ming
Zhang, Wenzeng
Zhang, Xianfeng
Zhang, Xiaopeng
Zhang, Xiaotong
Zhang, Xiaowen
Zhang, XinMing
Zhang, Xinyu
Zhang, Xionghu
Zhang, Xuehua
Zhang, Xuming
Zhang, Xu-Yao
Zhang, Yafei
Zhang, Yan
Zhang, Yanping
Zhang, Yimin
Zhang, Ying
Zhang, Yong
Zhang, Yongjun
Zhang, Yong-Mei
Zhang, Yuanzhi
Zhang, Yuting
Zhang, Yuzhong
Zhang, Zhongzhao
Zhang, Zixing
Zhao, Chihang
Zhao, Chunjiang
Zhao, Hui
Zhao, Huimin
Zhao, J. H.
Zhao, Jianzhang
Zhao, Kaichun
Zhao, Peng
Zhao, Qianchuan
Zhao, Vicky
Zhao, Wei
Zhao, Weisheng
Zhao, Xiangyong
Zhao, Yi
Zhao, Yindi
Zhao, Yong
Zhao, Zhan
Zhen, Lei
Zheng, Guilin
Zheng, Jane
Zheng, Jian-Bin
Zheng, Jihong
Zheng, Lihong
Zheng, Naiquan
Zheng, Rencheng
Zheng, Siyang
Zheng, Youbin
Zheng, Yuanyi
Zhi, Xiong
Zhiganov, S. N.
Zhong, Jiaofei (Fay)
Zhong, Xunyu
Zhou, Daiying
Zhou, Dengpan
Zhou, Donghua
Zhou, Fengfeng
Zhou, Fugen
Zhou, G. H.
Zhou, Huiyu
Zhou, Jun
Zhou, Liang
Zhou, MengChu
Zhou, Qian-Yi
Zhou, Qifa
Zhou, Qingli
Zhou, Xiao Dong
Zhou, Yu
Zhou, Yunshen
Zhou, Zhi
Zhu, Chun
Zhu, Congyong
Zhu, Guizhi
Zhu, Guoqiang
Zhu, Hao
Zhu, Hong-Hu
Zhu, Ji
Zhu, Ming
Zhu, Minghui
Zhu, Qing
Zhu, Weihong
Zhu, Xin
Zhu, Xu
Zhu, Yanhe
Zhu, Yinian
Zhu, Yiqun
Zhu, Zhongkui
Zhuang, Yanyan
Zielinski, Tomasz
Zimmerman, Patrick H.
Zingaretti, Primo
Zinnert, Julie
Ziolkowski, Robert
Zito, Domenico
Zou, Jie
Zou, Xiaotian
Zubia, Joseba
Zug, Sebastian
Zwyssig, Erich

